# *Drosophila* as a Model for Studying the Roles of Lamins in Normal Tissues and Laminopathies

**DOI:** 10.3390/cells14171303

**Published:** 2025-08-22

**Authors:** Aleksandra Zielińska, Marta Rowińska, Aleksandra Tomczak, Ryszard Rzepecki

**Affiliations:** Laboratory of Nuclear Proteins, Faculty of Biotechnology, University of Wroclaw, Fryderyka Joliot-Curie 14a, 50-383 Wroclaw, Poland; aleksandra.zielinska3@uwr.edu.pl (A.Z.); marta.rowinska@uwr.edu.pl (M.R.); aleksandra.tomczak@uwr.edu.pl (A.T.)

**Keywords:** *Drosophila melanogaster*, lamin Dm, lamin C, laminopathies, muscular dystrophy, phosphorylation, interactome, cellular stress

## Abstract

Nuclear processes are fundamental to the regulation of cellular, tissue, and organismal function, especially in complex multicellular systems. Central to these processes are lamins and lamin-associated proteins, which contribute to nuclear structure, gene expression, and chromatin organization. The discovery that mutations in genes coding for lamins and lamina-associated proteins give rise to rare disorders—collectively called laminopathies—has intensified interest in this field among cell biologists and medical scientists. While many practical and clinically relevant questions about phenotype development and potential treatments require mammalian models, key molecular mechanisms and interactions have also been effectively studied in both vertebrate and invertebrate systems. This review focuses on a discussion of *Drosophila* lamins, their major properties, functions, interactions and post-translational modifications, with comparison to mammalian lamins, and a discussion of the value of fly models in studies of lamins in muscle tissue development and function in comparison to mammalian lamin B-type and A/C-type. In this paper, we have discussed the overall impact of lamin Dm and lamin C level manipulations on overall phenotype, especially on larval and adult muscles. We have thoroughly discussed the conclusions, which may have been drawn from experiments with overexpression of lamin C mutants mimicking lamin A laminopathy mutations. We have presented and discussed the suggestion that the mechanisms underlying *Drosophila* muscle phenotype development are similar not only to human dystrophic laminopathies but also to classical human muscular dystrophies such as Duchenne muscular dystrophy and Hutchison–Gilford Progeria syndrome. We suggest that the activation of the stress response contributes to the laminopathic phenotype detected in *Drosophila*. Finely, this review discusses in depth the lamin Dm and lamin C interactomes, discrepancies between String-based interactome networks, and our map of interactomes based on manual verification of experimental data on *Drosophila* lamin interactions.

## 1. Introduction

The study of nuclear functions across different biological contexts has benefited greatly from a diverse array of animal model systems. These range from invertebrates such as worms (e.g., *C. elegans*) [1] and fruit flies *(D. melanogaster*) [2], as well as early vertebrates such as zebrafish (*D. rerio*) [3] and frogs (*X. laevis* [4], and *X. tropicalis* [5]). The study also extends to birds (*G. gallus*) [6] and, finally, to mammalian models such as mice (*M. musculus*) [7], rats (*R. norvegicus*) [8], guinea pigs (*C. porcellus*) [9], dogs (*C. familiaris*) [10], pigs (*S. domesticus*) [11], and human-derived cell models, including cultured patient cells and induced pluripotent stem cell (iPSC) systems [12]. Notably, human models have gained increasing importance in studies related to disease phenotype development and classical or gene/RNA therapy procedures.

*Diptera* flies, starting from *Chironomus* [13] and later including *Drosophila melanogaster*, have been used as an excellent model system for genetic and cell biology studies, and have been at the leading front of fundamental discoveries [14]. Historically, the first studies of DNA and chromosome structures began with *Chironomus* [15] and *Drosophila* [16]; for a review, see Ashburner et al. (2005) [17]. In fact, the very early studies of DNA, chromosome structure, puffs, and transcription (especially heat shock-induced) utilized *Drosophila* polytene nuclei from larval salivary glands. To date, *Drosophila melanogaster* has been widely used as a model organism in practically all aspects of biology and medicine [18]. This model has been used for neural system model studies [19], as a general model for neurodegenerative disorders [20], and specifically for Parkinson’s disease, as well as for cancer [21] and aging [22].

*D. melanogaster* has been used from the beginning as an excellent model to study nuclear structure, the organization and structure of chromatin, gene expression regulation, domain structure, and protein–DNA/chromatin interactions [23,24,25,26,27,28]. Furthermore, studies on karyoskeletal structures of cell nuclei and mitotic chromosomes, the nuclear matrix, and nuclear scaffold structures have been conducted since the inception of this research field [29,30,31,32,33]. Each of the animal model systems mentioned above has been thought to have specific genetic, metabolic, physiological, and/or ethical features making it a better or worse model, depending on the detailed purpose of the study one plans to conduct. Given the practically infinite combinations of scientific or clinical questions related to nuclear function across various animal model systems, it is not possible to address all the issues associated with the wide variety of available models. Once the *Drosophila* model system has been considered, a large variety of scientific questions may be addressed [1,2,3,4,6,12,34,35,36].

Our study aims only to discuss the usefulness of this model system in studies on the function of nuclear structural proteins such as lamins, lamina-associated proteins, nuclear envelope (NE) proteins, linker of nucleoskeleton and cytoskeleton (LINC) complex proteins, and other interacting proteins with proven structural or mechanical functions within the cell nucleus. This study focuses on evaluating the advantages and limitations of the fly model to study general mechanisms regulating nuclear processes, with attention to neuromuscular laminopathies. Laminopathies are a heterogeneous group of diagnosed human diseases, characterized by diverse phenotypes that affect various organs and tissues. These phenotypes are gathered together as they result from a common genetic background; namely, mutations in genes encoding lamins and lamina-associated proteins. As a significant portion of this category consists of genes encoding nuclear envelope proteins, including those linking the nuclear karyoskeleton to cytoplasmic cytoskeletal structures—such as LINC complex proteins, nesprins, and their associated complexes—this group of diseases is also referred to as envelopathies. The most well-known examples of laminopathies/envelopathies include Emery–Dreifuss muscular dystrophies [EDMD1-*EMD* gene mutations (EDMD1, OMIM #310300), EDMD2-*LMNA* gene mutations (EDMD2, OMIM #181350), and other EDMDs (e.g., EDMD3, OMIM #616516, EDMD4, OMIM #612998; EDMD5, OMIM #612999; EDMD6, OMIM #300696, EDMD7, OMIM #614302)]; autosomal dominant leukodystrophy (ADLD, OMIM #169500, gene *LMNB1*); Charcot–Marie–Tooth disease, type 2B1 (CMT, OMIM #605588, gene *LMNA*); Hutchinson–Gilford progeria syndrome (HGPS, OMIM #176670, gene *LMNA*); acquired partial lipodystrophy, also called Barraquer–Simons syndrome (APLD, OMIM #608709, gene *LMNB2*); limb–girdle muscular dystrophy (LGMD OMIM #253600, gene *CAPN3*); Pelger–Hue’t anomaly (PHA OMIM #169400, gene *LBR*); Greenberg hydrops-ectopic calcification-moth-eaten skeletal dysplasia (HEM/GRBGD, OMIM #215140, gene *LBR*); and Buschke–Ollendorff syndrome (BOS OMIM #166700, gene *LEMD3*).

The *Drosophila* model may be useful in general studies of mechanisms that are potentially affected by mutations in genes relevant to particular diseases, as respective genes that have similar or identical functions may also be encoded in the fly genome. Moreover, certain genes in the fly genome exist as single members of the family. *Drosophila’s* second obvious advantage is its fast life cycle, as well as the availability of libraries of genetically modified lines for overexpression, knockdown, or knockout, potentially in a tissue-specific manner; availability of polytene and polyploid nuclei; easy collection of samples at various developmental stages; and minimal ethical issues [2,37,38,39].

## 2. Lamins—General Introduction

Lamins are essential for the structural organization of metazoan cell nuclei, playing both direct and indirect roles in maintaining proper nuclear architecture. They are crucial for nuclear mechanical stability, mechanosensing, and mechanotransduction [40]. In addition to these structural and mechanical roles, lamins are involved in chromatin organization and positioning, the regulation of gene expression, replication, transcription [41], and splicing [42]. They also contribute to the distribution of nuclear pore complexes (NPCs) [43], the linkage between the karyoskeleton and cytoskeleton, and interactions with the extracellular matrix (ECM) [38]. Furthermore, lamins participate in intracellular transport and regulate signaling pathways between the ECM, cytoplasm, nucleus, and chromatin [44]. Lamins are considered to be the evolutionally oldest group of intermediate filament proteins and are located inside the cell nucleus in contrast to all other groups of intermediate filament proteins, which are exclusively cytoplasmic. Lamins are generally divided into two classes—namely, B-type lamins and A-type lamins—based on their amino acid residue sequence, transcript processing, maturation, and properties. While lamins are generally located at the inner nuclear membrane (INM) and at the nucleolus/nucleoplasm border, there is a fraction of so-called “soluble” (less polymerized) lamins, which are localized in the nucleoplasm [37,40,45,46]. Lamins, together with interacting proteins—both intranuclear and those associated with the NE—form the nuclear lamina (NL) and intranuclear filament network [47,48]. This karyoskeletal network of interactions, which is modulated by post-translational modifications, has a direct impact on a plethora of major nuclear processes such as chromatin organization, replication, regulation of transcription, cell cycle regulation, and signaling [49,50].

In the mammalian genome, three genes encode both lamin types. *LMNA* is a gene coding for multiple proteins by alternative splicing (lamin A, lamin C, lamin A(Δ10), lamin C2; all A-type lamins). Meanwhile, B-type lamins are encoded by *LMNB1* and *LMNB2* genes. The only protein that is a product of the *LMNB1* gene is lamin B1, while the *LMNB2* gene encodes lamin B2 and B3 [51]. Lamin B3 and lamin C2 are germline- and (probably) mammalian-specific [39].

In the genomes of certain vertebrates, such as amphibians, teleost fish, and birds, four genes code for lamin proteins; namely, lamins B1 (LI), B2 (LII), B3 (LIII), and lamin A. Lamin B3 is only expressed in the early embryo and oocytes, whereas lamin A is not spliced to the lamin C protein. Additionally, there are three products resulting from the alternative splicing of lamin B3; namely, lamins B3a (LIIIa), B3b (LIIIb), and LIV [38,39]. Members of each lamin class may have overlapping functions in somatic cells, contributing to complications of vertebrate model systems when the properties of particular lamins are being studied.

In *C. elegans*, there is only a single gene coding for Ce-lamin, which exhibits properties characteristic of both A- and B-type lamins. *Drosophila melanogaster* differs from the *C. elegans* model, as the former has two genes coding for two lamin types. The *lamin C* gene encodes an A-type lamin protein, which is called lamin C. Fly lamin C demonstrates a mixture of lamin A- and C-type features. Meanwhile, a B-type lamin protein called lamin Dm is encoded by the *lamin Dm* gene [2]. Therefore, the *Drosophila* model makes it much easier to study the complex functions of particular B- and A-type lamins, compared to vertebrates, in which multiple genes encoding lamins and alternative splicing generate a variety of lamin proteins [37].

It should be pointed out here that various new names, such as lamin Dm_0_, lamin Dm0, Dm0, Dm_0_, lamin B, and Lam for the lamin Dm protein and gene have been introduced recently (according to FlyBase, FB2024_06). Initial reports named the protein lamin Dm, in general, or lamin Dm_1_, Dm_2_, D_mmit_, or lamin Dm_0_ (direct translation and direct in vitro translation product, *in statu nascendi*/unphosphorylated) when specific interconverting forms were described. The new names Lam and LamC for lamin Dm and lamin C, respectively, may cause some confusion when both lamin Dm and lamin C are considered subjects in the same study, especially as typing mistakes can occur. Therefore, to avoid confusion, we shall use the traditional names for fly lamin proteins—namely, lamin Dm and lamin C—in this review.

All lamins have the characteristic structure of intermediate filament (IF) proteins, which contain a small amino-terminal (N-terminal), non-helical head domain, a long coiled-coil central rod domain, and a large carboxy-terminal (C-terminal) tail domain. The rod domain contains four alpha-helical coils connected by three linkers [52,53,54]. However, some researchers have suggested that the second domain and linker should be treated as one [55,56]. The C-terminal globular domain consists of conserved amino acid segments forming an immunoglobulin-like fold, which is a structural motif that interacts with DNA and other nuclear proteins. B-type lamins differ from A-type lamins in that they remain permanently farnesylated, while lamin A lacks the anchor. In the tail domain of almost all nuclear lamins (excluding lamin C), a carboxy-terminal CaaX box (C—cysteine; a—aliphatic amino acid; X—any amino acid) is located for farnesylation of the cysteine. After lipid addition to the cysteine residue, the last three amino acids are cleaved off and methylation occurs. As mentioned above, B-type lamins are permanently farnesylated, while lamin A loses the farnesylated tail via proteolytic cleavage [47]. The C-terminal domain of lamins includes a nuclear localization signal/sequence (NLS), which is required for nuclear import [57].

Lamins are organized from monomers into dimers and tetramers via the N- and C-terminal parts of the rod domain. Moreover, lamin dimers associate in a head-to-tail manner, further forming thicker filaments. Recent studies have shown that lamin A and lamin B networks are organized as 3.5 nm thick filament meshwork in mammalian tissue-cultured cells [45]. Data from different models have reported lamin filaments ranging from 10 nm thick filaments up to 50–200 nm nuclear lamina filament meshwork. The most regular lamin filament structure is assembled in *Xenopus* oocytes, with different networks for B- and A-type lamins under experimental conditions following the ectopic expression of the respective proteins [58,59].

Lamins are involved in a wide range of cellular processes and have been implied to interact with a large number of proteins, including both structural proteins as well as enzymes and regulatory proteins (e.g., second messengers, kinases, and phosphatases) [60,61]. Lamins can be described as a sort of hub or docking platform (a skeleton network) for multiple relatively transient interactions, as well as a framework for important nuclear processes. These interactions, along with the assembly of lamins, are regulated by lamin post-translational modifications, changes in partner modifications, or both. Although our knowledge of the lamin proteome network has been expanding rapidly, as well as knowledge regarding modulation of the lamin interactome by extracellular signaling and during the cell cycle, the best-known protein partners so far are other structural proteins present in the cell nucleus, such as other nuclear lamina and nuclear envelope proteins [59,62,63].

The most important and best-known lamin-interacting proteins are LEM (LAP-Emerin-MAN1) domain proteins, linker of nucleoskeleton and cytoskeleton complex (LINC complex) proteins, components of the nuclear basket of nuclear pore complexes (NPCs), and a range of soluble intra-nuclear regulatory proteins and enzymes [64,65]. The LEM-domain protein family is defined by the presence of a conserved LEM domain and at least one transmembrane (TM) segment. Some members or alternatively spliced isoforms additionally contain LEM-like motifs and other functional domains required for interactions with nuclear lamina components or soluble nucleoplasmic factors. The canonical LEM domain mediates binding to Barrier-to-Autointegration Factor (BAF), which in turn anchors these proteins to chromatin through interactions with histones and DNA. In humans, the family includes multiple LAP2 isoforms, emerin, and MAN1. Most LAP2 proteins contain a transmembrane domain, except for LAP2α and ANKLE2. LAP2α is involved in the maintenance of the soluble intranuclear lamin A fraction and takes part in the regulation of cell cycle progression and differentiation through its interaction with the Rb/E2F complex—a key regulator of the cell cycle and transcription [66]. ANKLE2 is essential for the regulation of lamins and chromatin reassembly after mitosis. The LINC complex is composed of SUN and KASH domain proteins, spans through the inner and outer nuclear membrane, and connects directly and indirectly to cytoskeleton elements (including actin polymers, microtubules, and centrosomes) as well to the cytoplasmic intermediate filament protein network. This complex, as a nucleoskeleton–cytoskeleton interconnector, is responsible for maintaining the proper tissue-specific location of cell nuclei, nuclear transport, mechanotransduction, and mechanosensing [67,68,69,70].

## 3. Fly Lamin Dm Structure and Function

Fly lamin Dm is a canonical B-type lamin. A gene for lamin Dm was cloned and sequenced in 1988, which encodes two mRNAs that are translated into a single protein, at that time called lamin Dm_0_ polypeptide. This protein serves as a precursor for two lamin Dm forms: lamin Dm_1_ and Dm_2_ [71]. As a direct product of translation, lamin Dm_0_ polypeptide is immediately converted by phosphorylation to lamin Dm_1_ polypeptide, and then, under certain conditions, its further phosphorylation generates lamin Dm_2_ polypeptide. The two latter polypeptides represent the interphase lamin Dm fraction [72].

**Figure 1:**
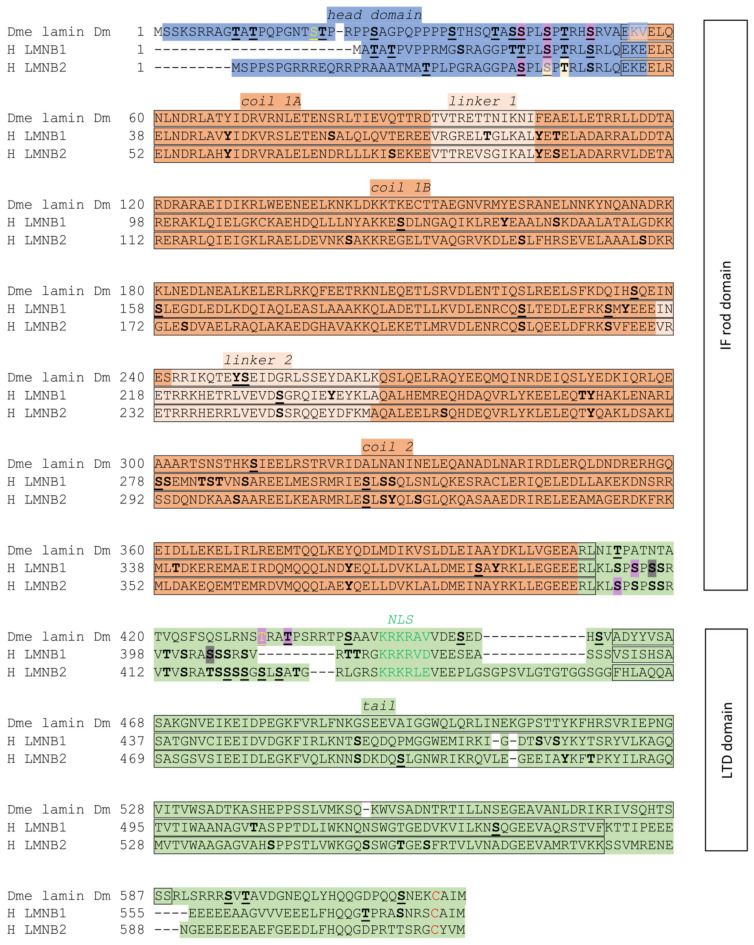
Multiple sequence alignment for B-type lamins from *Drosophila melanogaster* (Dme)—lamin Dm and *Homo sapiens* (H)—lamin B1 and lamin B2, with a particular focus on lamin structural elements and the similarity between their sequences. Alignment prepared using Clustal Omega (clostalo 1.2.4 online version; https://www.ebi.ac.uk/Tools/msa/clustalo/ (accessed on 14 March 2022) with the default algorithm). Sequences, structure regions, and motifs were downloaded from the UniProt and iPTMnet phosphorylation motifs databases (validated on 15 February 2021). Description of color marks and symbols used in alignments: **S, T,** and **Y** (bolded and underlined) amino acids depict the phosphorylation site according to the UniProt and iPTMnet databases. **S, T,** and **Y** (bolded) amino acids depict the phosphorylation sites according to iPTMnet with additional data sources: phospho.ELM, PhosphoSitePlus, and Human Protein Reference Database (HPRD). S and T (yellow and underlined)—experimentally confirmed however not annotated in the UniProt database; Red C—S-farnesyl cysteine site. Phosphorylation sites: cyclin-dependent kinase 1 phosphorylation site (Cdk1)—purple; cyclin-dependent kinase 2 phosphorylation site (Cdk2)—beige; and protein kinase C beta type phosphorylation site (PKCβ)—gray.

Lamin Dm polypeptide interconversion from Dm_0_ to Dm_1_ and further to Dm_2_ was earlier detected under growth and heat shock conditions and during in vitro translation experiments coupled with radioactive phosphate labeling [73]. The interconversion between newly synthesized lamin Dm_0_ and lamin Dm_1_ and Dm_2_, as well as Dm_mit_, was reported [73] in nuclear matrix preparations, including those from heat-shocked cells [74] as well as during oogenesis, embryogenesis, and mitotic entry [75]. The above-mentioned papers also demonstrated that the conversion occurs in a phosphorylation-dependent manner. Stuurman et al. (1995) [76] later described phosphorylation site mapping using the antibodies ADL84 and ADL67, while LC-MS/MS allowed for the identification of S25, S595, and S45 as being phosphorylated in vivo [77]. Later on, additional in vivo phosphorylation sites were identified on S19 and T597 [77]. Different possible functions (including solubility) of single phosphorylation sites in lamin Dm in vitro have been demonstrated in the study of Zaremba-Czogalla et al. (2012) [52]. Radioactive orthophosphoric acid labeling identified up to three phosphorylated amino acid residues on lamin Dm in vivo [78]. Lamin Dm undergoes farnesylation as is characteristic of B-type lamin [79] and phosphorylation at specific sites: during mitosis at Cdk1-specific sites, called mitotic sites (S45; T432/435), and various interphase sites [77,80], each suggested to have different effects on the properties of lamin Dm [52]. It is known that freshly translated lamin Dm protein is called Dm_0,_ which reportedly lacks phosphorylation. Phosphorylation then transforms this protein into the Dm_1_ form, while the lamin Dm_2_ protein isoform arises from further phosphorylation [78] of at least one residue localized at S25 [76] and/or S19 [77]. The ratio between lamins Dm_1_ and Dm_2_ varies, in a manner depending on many factors such as cell confluency, the embryonic stage, cell cycle stage, and so on; when calculated based on Western blot densitometries, this ratio was found to typically vary from more than 3:1 up to 1:3 or higher, respectively [81]. Recent studies using optimized protocols for LC-MS/MS analyses discovered many in vivo phosphorylation sites on lamin Dm and mammalian B-type lamins (see Figure 1). Moreover, Dm_1_ and Dm_2_ are insoluble in low-salt buffers in contrast to lamin Dm_mit_, which is converted from the interphase isoforms by precise phosphorylation and dephosphorylation events. Derivatives of lamin Dm are present in all cell types, except for mature sperm [73,78].

Lamins undergo many other post-translational modifications such as myristoylation, ubiquitination, acetylation, sumoylation, proteolytic cleavage, and phosphorylation [62]. The last modification is very important for lamins, as their functions notably depend on phosphorylation. During nuclear envelope breakdown, extensive phosphorylation of lamins occurs, leading to the solubility of the protein; notably, this mechanism makes nuclear disassembly and assembly possible [82]. It should be pointed out here that early embryonic mitotic cycles can be described as semi-closed mitoses, with only fractions of NE depolimerases in polar regions. The rest of fly mitotic divisions are typical open mitotic cycles. In the head and tail domains of lamins, there are two localized mitotic phosphorylation sites; these conserved motifs are significant for the polymerization and depolymerization of all lamins [52]. For a deeper discussion on the role of phosphorylation and the functions of lamins, see Machowska et al. (2015) [60]. Recent studies have demonstrated that phosphorylation of lamin Dm S25 is reversibly induced upon entry into heat-shock response, which is correlated with increased lamin Dm–topoisomerase II (Top2) interaction, modified solubility of both proteins, increased efficiency of nucleic acid binding, and increased binding to RNA by both proteins [83].

The *Drosophila* mature lamin Dm is a nuclear protein that has 622 amino acid residues [38,56]. In comparison, human lamin B1 consists of 586 residues, while lamin B2 comprises 620 residues. The head domain of lamin Dm spans 55 residues, whereas that of human lamin B1 has 33 residues and lamin B2 has 48 residues. There are slight differences in the rod domain sequence (lamin Dm: 354; B1 and B2: 352). Notably, the tail domain of lamin Dm contains 211, whereas lamin B1 has 200 residues and lamin B2 has 220 residues. This region exhibits the most significant differentiation in terms of length. Despite these subtle length variations, considering the distant evolutionary relationship between fruit flies and humans, the B-type lamins show remarkable similarities. Alignment of B-type lamins from *Drosophila* and humans revealed that lamin Dm shares 36.99% amino acid identity with lamin B1, while the identity with lamin B2 is slightly lower at 36.96%. Importantly, *D. melanogaster* and *Homo sapiens* B-type lamins share a common structural framework and possess identical functional domains. Specifically, the *Drosophila* lamin Dm CaaX sequence likely facilitates the transport of lamin Dm to the inner membrane. This modification is also necessary to maintain the mitotic B-type lamin fraction associated with mitotic membranes/vesicles, both at the mitotic spindle envelope and dispersed through the rest of the cell [37,56,84].

Within the head domain of lamins, there is at least one phosphorylation site motif—namely, the N-terminal mitotic site—that is recognized and phosphorylated by Cdk1 during mitosis. The phosphoacceptor serine residue in this conserved motif SPTR is conserved across species. For the fruit fly lamin Dm, the position of this residue is S45. For human lamin B1, it is S23, while that in B2 is S37 (see Figure 1). Zaremba-Czogalla et al. (2012) [52] revealed, through an in vitro experiment, that pseudophosphorylation of S45 caused insolubility of the whole protein [52]. In the head domain, there is a conserved motif SPL. Within this motif, in lamin Dm, the S42 serine residue is a phosphoacceptor. For human lamin B1, this motif is absent; however, for B2, the phosphoacceptor is at S34 [38]. There is also another characteristic feature at the lamin head domain that is conserved among species and types: the “TP” motif. It is located at T20 and P21 residue in lamin Dm and, in humans, at T5 and P6 in lamin B1 and T23 and P24 in lamin B2. Furthermore, A-type lamins also contain this motif, which is present just before the N-terminal Cdk1 site. Sites identified as phosphorylated but with lower frequency include T3 and T5 in human lamin B1; T23 in lamin B2; and T10 and T12 in fly lamin Dm. These sites are predicted to be phosphorylated by (PK)A, PKC, Cdk5, Gsk3, and ERK1 kinases. Additional phosphorylation motifs present in fly lamins differentiate them from vertebrate lamins and may influence their secondary structure and solubility properties. There is also a conserved PKC/PKA site located upstream of the N-terminal “mitotic” site. The phosphoacceptor serine residue is S50 in lamin Dm. In human lamin B1, its localization is S28, and in lamin B2, it is S42. Additionally, in *Homo sapiens* lamin B1, there is an SR motif, as a serine residue that could also be phosphorylated. The lamin Dm head domain might create a hairpin-like structure through interaction with the rod domain. The same region in the head domain contains S19, T20, and S25 phosphorylated in vivo, point mutations that change the solubility properties of this protein [60].

Additionally, there is some evidence of phosphorylation sites within the rod domain, both in *Homo sapiens* [85,86,87] and *D. melanogaster* [71,88] B-type lamins; however, these are less frequent than in head and tail domains. This could be an interesting field for further investigation.

The tail domain of lamin proteins contains crucial sequences that are conserved across all lamin isoforms. Notably, this region includes an essential and conserved C-terminal Cdk1 phosphorylation site, which plays a critical role in the polymerization and depolymerization of lamin proteins. In *Homo sapiens*, this site corresponds to serine residues S391 and S393 in lamin B1, and S405 and S407 in lamin B2. The *D. melanogaster* lamin also contains a C-terminal Cdk1 phosphorylation site, but it is not a canonical one. This site is probably located at T432 or T435, where the second one is possibly stronger and could be phosphorylated by the whole group of Cdk protein kinases. Pseudophosphorylation of T435 makes this protein soluble, which changes the localization to nucleoplasmatic. Surprisingly, single-residue phosphorylation did not influence its chromatin binding properties, while the nuclear import of lamin Dm was blocked after the pseudophosphorylation of T435. In humans, lamin B1 has been identified as possessing a phosphorylation site for PKC, which is S395. This is characteristic for both humans and *Drosophila* lamins [60]. Furthermore, this Cdk1 site is among the conserved TPSR motifs (S/TRAS/T for other organisms) [52]. The previously mentioned conserved motif S/TRAS/T is located in the tail domain, and, in it, there is a serine residue in lamin B1, S405, which is phosphorylated by PKC (Figure 1).

Typically, the phosphorylation of residues located within the head and tail domains of lamins promotes their depolymerization, especially those located next to the rod domain. This could result in disruption of the nuclear lamina network. Furthermore, phosphorylation of B-type lamins in the Cdk1 site within the tail domain results in the partial disassembly of lamin fractions. Moreover, there are other phosphorylation sites and motifs localized on lamin Dm that are comparable with vertebrate lamins—in particular, within this group, with human lamins [52,60].

Lamins contain one 14-3-3 protein domain-binding motif. This protein can bind to a large number of proteins in a phosphorylation-dependent manner and plays a critical role in the regulation of the cell cycle, and among other functions, it is involved in the modulation of locomotion and the functioning of phosphoproteins. In lamin Dm, the sequence binding the 14-3-3 protein is R22PPS, which is located in the head domain; similarly, in the human B1-type, this is the R10MGS sequence [60,89]. Please note that S25 has been identified as phosphoacceptor in vivo [76,80].

The functional importance of the lamin head domain has been demonstrated through direct in vitro experiments. This domain takes part in head-to-tail polymerization [52,90,91]. N. Stuurman et al. (1996) showed that phosphorylation in vitro by the cAMP-dependent kinase or Cdk1 at lamin Dm residues (S42 and S50, respectively) inhibits head-to-tail polymerization [92]. Moreover, lamin Dm filaments assembled in vitro could be disassembled upon phosphorylation by Cdk1 or PKA kinase. Additionally, polymerization of native or bacterially expressed and purified lamin Dm is dependent on its connection with small heat-shock proteins and can be restrained by site-specific antibodies. Unexpectedly, lamin Dm does not need to be fully polymerized to bind directly to DNA or chromatin components [52,60]. It has been shown, in in vivo research, that lamin Dm associates with nucleic acid only during interphase [93], whereas in vitro lamins bind to the telomeric and centromeric sequences, or scaffold/matrix attachment regions (S/MARs) [93,94]. Furthermore, in vivo, lamins bind chromatin directly or indirectly [52]. The S/TRAT/S sequence, as well as the NLS, localized in the C-terminal domain of both mammalian A/C lamins and lamin Dm, seems to play a special role in chromatin and DNA binding. This C-terminal fragment of lamin Dm binds to histones H2A and H2B. In lamin Dm, mutation of the S431TRAT sequence prevented it from binding to chromosomes in vitro [60]. The role of farnesylation and other lamin Dm domains have been studied in projects using a fly strain called *Lamin Dm^A25^*. This strain carries a *lamin Dm* gene sequence with C-terminal deletion, resulting in the synthesis of truncated lamin Dm protein lacking the terminal sequence CAIM (farnesylation motif), resulting in non-farnesylated lamin Dm protein synthesis. Although their results were not fully comparable, two studies have demonstrated the necessity of farnesylation in the anchorage of lamin Dm to the INM [95,96]. The first report indicated that CaaX-motif-deleted lamin Dm formed a nuclear envelope-independent layer associated with the surface of polytenic chromosomes, while lamin C retained NE association. Lamin Dm association with chromatin induced increased levels of repressive histone modification markers [95,96].

Deletion of Ig-fold prevented mutant lamin Dm from associating with chromatin [95]. The second report confirmed the association of a lamin Dm CaaX-motif deletion mutant with polytene chromosomes and the normal NE/peripheral association of lamin C, heterochromatin protein 1 (HP1a), and H3K9me2- and H3K27me3-associated chromatin. In proventriculus nuclei, the fluorescence intensity of peripheral chromatin decreased while chromatin in central regions presented increased fluorescence intensity [96]. These observations suggest that lamin Dm and lamin C may form (at least partially) independent networks. The separation between lamin Dm and lamin C fractions has been demonstrated during mitosis, where, in somatic cells, the lamin Dm fraction associates mostly with the so called mitotic spindle envelope, probably associated with membrane vesicles (membranes) not only in *Drosophila* but also in mammalian cells as demonstrated for lamin B1. Lamin C, similarly, to mammalian A/C lamins is dispersed during mitosis [97,98,99].

Several reports have indicated that newly synthesized populations of lamin Dm and lamin C form independent networks [100]. The presented data on lamin Dm forming a layer on polytenic chromosomes independently of the NE may not be fully comparable with those from vertebrate cells, indicating that B-type lamin is connected directly to the nuclear membrane through the CaaX motif, which makes A-type lamin dependent on the other (B-type) lamin [101,102,103]. An explanation for this observation might be due to the animal model (or, more probably, the polyploidity/polythenity of fly cells serving as a model) being compared to the tissue culture model used in vertebrate-based research. In any case, it has been demonstrated that lamin Dm and lamin C form independent networks at the nuclear lamina in *Drosophila* [100].

Interestingly, overexpression of wild-type lamin Dm or lamin Dm fused to the GFP protein in cells induced the formulation of lobulated nuclei and multilayered membrane assemblies, similar to overexpression of mammalian B-type lamins and expression of progerin, while overexpression of lamin C had no effect. However, when mutant lamin C with the CaaX motif inserted was overexpressed, this induced similar lobulation of nuclei and multilayered membrane assemblies [104]. Furthermore, overexpression of GFP with the NLS and CaaX motif caused nuclear lobulations and intranuclear membrane stacks [105]. Similar lobulations and nuclear membrane stacks were detected when B-type lamin or lamin Dm were overexpressed in HeLa and *Drosophila* tissue-cultured cells, respectively [52]. This CaaX motif-associated phenomenon raises a question regarding whether lobulated nuclei in HGPS patient fibroblasts, expressing progerin (lamin A mutant retaining CAAX motif), are the specific feature of HGPS, or just the simple effect of large amounts of progerin accumulated at the nuclear membrane, or both; that is, the accumulation of CaaX protein (progerin) causes lobulation, which then causes further abnormalities in the chromatin structure and gene expression profile.

## 4. Role of Lamin Dm in the Development and Tissue Functions of *Drosophila*

To study the specific roles of lamin Dm, many experiments have been conducted, mainly based on loss-of-function phenotypes through the use of the knockdown or knockout method; however, several overexpression studies have also been reported. The interpretation of the results of such studies seems to be a little complicated in *Drosophila*, due to the high level of lamin Dm deposited in oocytes, which seems to last to at least the second-instar larval stadium in Western blot (WB) studies and also persists in several tissues at the third-instar larvae, as detected through IF studies. Generally, fly mutants *Lam^14^* and *misg^sz18^/Df* are more effective in removing lamin Dm protein from larval tissues, especially at the third-instar larval level, when compared to the *Lam^P^* strain. The lines *Lam^14^/CyO* are 100% fertile, *Lam^P^* and *Lam^14^/Lam^P^* are sterile and reach imago with about 50% efficiency, and *misg^sz18^/Df* and *Lam^14^/Df* survive up to late pupa with about 75% efficiency. A similar effect was detected in the case of *Lam^14^* and *Lam^9^*, but with slightly lower efficiency [43,84,106]. In another study presenting the effects of other lamin Dm null mutants together with the *Lam^P^* strain on adult flies, some differences in survival rates were reported (especially regarding the *Lam^P^* strain). As no data on the level of lamin Dm were provided, it is difficult to compare and discuss this contradictory report [107]. The phenotypes and molecular phenotypes demonstrated in lamin Dm deficiency studies are presented in Table 1. The general conclusion from the above experiments is that the larval and early pupa phenotype strength depends on the level of remaining lamin Dm protein in particular tissues, while the extent of late pupa and imago phenotypes depends rather on the level of lamin Dm in imaginal discs and adult tissues. For the strength of the larval phenotype, in general, and in muscle tissues, fat body, imaginal discs and the CNS, the critical issue is the efficiency of lamin Dm removal in knockout models and the efficiency, specificity, and strength of drivers being used for GAL4-mediated knockdown experiments.

The complex development of *Drosophila* has positive and negative consequences from the researcher’s perspective. The advantage of this model is that it allows for the study of embryonic and larval development, encompassing all larval tissues including larval muscles. Additionally, it provides the opportunity to investigate the development of the adult fly and associated phenotypes; however, a drawback stems from the fact that most adult tissues start to develop from larval imaginal discs. Another complication is the histolysis during pupation. Manipulations of protein levels and exogenous expression/overexpression of proteins or protein mutants that might have affected histolysis would have an effect on the efficiency of eclosion, the final survival of adults, or the adult phenotype. Therefore, the phenotype and gene expression/protein content profile of imaginal discs develop during the larval stage but may affect pupation and imago development, leading to the interplay of the lamin Dm level in developing/growing adult tissues. This interplay may complicate the interpretation of data in adult fly-based studies.

The very first report on the biological role of lamin Dm in vivo was put forward by Lenz-Böhme et al. (1997) [84]. The authors analyzed a fly mutant with a reduced amount of lamin Dm, obtained by inserting the P element into the first intron of the *lamin Dm* gene (*Lam^P^* strain). First of all, the movement of larval mutants was slower, and they needed more time in the test of righting response. Flight ability was lost in some mutants, and the time of development was delayed up to three days, with only 5–10% of the balanced mutants reaching adulthood. Female and male flies were sterile, with motionless sperm and aberrant female gonads, associated with abnormal oocytes and atypical nuclear morphology with clustered NPCs. Interestingly, NPCs often differed from the normal octagonal symmetry. It should be pointed out that mutant adult fly homozygotes expressed detectable amounts of lamin Dm protein in Western blot experiments [84].

Lamin Dm seems to provide proper egg polarity and development of the tracheal system in embryos, as demonstrated by Guillemin et al. (2001) [106]. Germline mutant clones (*misg^18^*) with zygotic loss-of-function mutations greatly reduced lamin Dm in nurse cells and oocyte nuclei but presented normal levels of lamin Dm in epidermal cells of the oocyte. The mutants exhibited impairments in the dorsoventral axis formation of the oocyte, associated with mislocalization of *gurken* transcripts and Gurken proteins. Proper localization of *gurken* transcripts requires accurate migration and anchoring of the oocyte nucleus, as well as a correctly polarized microtubule cytoskeleton. These observations suggest that lamin Dm is essential for tethering the microtubule network to the nuclear envelope, thereby contributing to nuclear positioning and the establishment of oocyte polarity. Additionally, the direction of the cytoplasmic extensions was disrupted in terminal cells of the tracheal system. This result might help to explain the pathogenesis of human diseases linked to lamin mutations, especially in those that are not easily explainable due to abnormalities in nuclear organization or structure [106]. Both reports’ data [84,106] suggest that lamin Dm is important for proper nucleus transport to proper locations during oocyte maturation, probably by maintaining a proper LINC complex distribution and connection to microtubule networks, an important function in supporting proper distribution and assembly of NPCs.

In a recent paper, Penfield and Montelli (2023) [110] reported another function of lamin Dm in oocytes: lamin Dm facilitates collective border cell migration, as its lack affects nuclei structural integrity and hinders the expansion of leading protrusions, thus impeding border cell movement. This study confirms the thesis of aberrant linkage between nucleus structures (NL/LINC) and microtubule networks.

Osouda et al. (2005) [43] investigated the role of B-type lamin in *Drosophila melanogaster* by creating two new null mutants with the use of a P-element (*Lam^14^* and *Lam^4643^*) and compared new mutants’ phenotypes with previously described ones. The new mutants retained lamin Dm staining up to the second-instar larval stage, as well as positive IF staining in several tissues and organs at the third stage and in early pupae (e.g., central nervous system, CNS). Surprisingly, the new phenotype showed a lack of abnormalities in nuclear structure, and only an irregular NPC distribution was observed. This might have been attributed to retained lamin Dm presence in nuclei of most tissues during larval development till the third-instar larvae stage. Furthermore, the researchers noticed that there was no compensation for lamin Dm by lamin C. The fly phenotypes indicated abnormal organ development, including strongly pigmented and accumulated red material in compound eyes and a partially semi-transparent exoskeleton in the ventral abdominal part. Moreover, the development of both female and male gonads stopped at an early stage. This phenotype confirms earlier suggestions that the necessity of lamin Dm in gonad development results in dysfunctional oocytes and sperm. Retarded growth was also observed in the central nervous system. In contrast, the digestive tract (ventriculus) showed over-proliferation. This last observation might not apply to the entire digestive system and/or the entire population of cells, as a later report demonstrated that knockout of lamin Dm or Kugelkern retained the proliferation of intestine stem cells (ISCs), while overexpression of each of the proteins strongly suppressed proliferation [111]. The Kugelkern protein contains the CaaX farnesylation motif in its C-terminus and a putative coiled-coil domain in the N-terminus. This protein is necessary for nuclei elongation, proper chromocenter assembly, and the expression of early zygotic genes. Overexpression induces membrane growth [112]. It is possible that the difference in effects was related to the lower level of lamin Dm in *Lam^14^* and *Lam^4643^* mutants compared to the two above-mentioned mutants. Further research showed that hypertrophy of the ventriculus was related to a reduction in the protein ecdysteroid hormone receptor B1 (EcRB1), which takes part in pupal organogenesis. The authors suggested that the results of this work showed that lamin Dm more likely plays a role in tissue differentiation, rather than affecting nuclear morphology. It has been mentioned that the most evident aberration was in terms of the NPC distribution, which corroborates results obtained when working with human lamin B1 with a rod-domain deficiency [43,102]. It is possible that the differences between phenotypes described in the above mutants, *Lam^P^*, *misg^18^* and *Lam1,4* and *Lam^4643^* [43,84,106], might be explained by different amounts of retained lamin Dm in analyzed tissues. This hypothesis might be supported by comparison of phenotypes and lamin Dm levels in tissues–see Table 1. Please note that the survival/lethality phenotype for the same mutant analyzed differs slightly between reports.

Another function of lamin Dm was discovered when immunosenescence was analyzed in the fly model by Chen et al. (2014) [108] in an attempt to assess the aging of immune organs. This is connected with age-associated loss of B-type lamin, which is connected with heterochromatin loss as well as the expression of genes involved in the immune response. *Drosophila* and mammalian lamins are known to have domains enriched for immune response genes, such that the resulting loss could lead to immunosenescence during the aging of higher organisms such as mammals and humans. This research investigated the consequences of *lamin Dm* expression silencing in a tissue-specific manner in comparison with old flies. Knockdown was obtained using the Gal4-UAS system with fat body and midgut-specific promoters for flies. The research demonstrated that depletion of lamin Dm in adult flies’ fat bodies resulted in changes in the expression of hundreds of genes. Surprisingly, those genes overlapped with those that had been changed upon fat body aging. Furthermore, lamin Dm depletion led to increased IMD (immune deficiency) signaling in the absence of infection. Furthermore, Chen et al. (2014) [108] have reported a decrease in levels of heterochromatin markers (HP1a, H3K9me3) in fat bodies of both aged flies and lamin Dm knockdown mutants, which implies gene and cell type-dependent suppression of genes responsible for immune defense. A lamin Dm loss-associated reduction in heterochromatin has been reported previously by Bank and Gruenbaum (2011) [53], as well as Dechat et al. (2008) [113], and further discussed by van Steensel and Belmont (2017) [114]. Nevertheless, the number of genes affected by tissue-selective GAL4-RNAi-mediated depletion of lamin Dm—complete depletion (over 1600 upregulated and over 800 downregulated)—suggests that lamin Dm plays a rather general role in gene expression regulation, and the deregulation of immune response genes is only a small part of this general mechanism.

Another considered role of lamin Dm is the regulation of nuclear actin polymerization. Dopie et al. (2015) [109] performed genome RNAi screening in fly S2 cells with the goal of finding proteins that play a role in nuclear actin import or polymerization. The results were analyzed by “rescuing” the “nuclear actin bar” appearance via exportin 6 reduction. Depletion of lamin Dm with exportin 6 resulted in a signal from a heightened amount of nuclear actin. A similar role was assigned to Nup98. Similar results have been obtained by Ho et al. (2013) [115] in mammalian cells, but for the A-type lamin. Thus, we may safely conclude that lamin Dm and Nup98 are necessary for proper NPC function in active actin export from the cell nucleus.

Lamin Dm and Nup107 also play roles in proper meiosis I during *Drosophila* male meiosis, as the knockdown of both proteins was found to affect cytokinesis. Furthermore, during meiosis I, lamin C was completely disassembled before metaphase and its components were dispersed throughout the cytoplasm, while lamin Dm, or at least its fraction, remained associated with the spindle envelope. This suggests that a fraction of mitotic vesicles/mitotic membranes containing lamin Dm have been recruited to the mitotic spindle envelope (or chromatin) via interactions with Nup107 protein by a not fully known mechanism since depletion of Nup107 mislocalizes lamin Dm staining to more or less dispersed aggregates. It is possible that this lamin Dm/Nup107 complex location can be mediated by conservative mechanisms of nuclear import via the Run protein/importin complex, released next to meiotic chromosomes by interaction with Rcc1 protein. Lamin Dm and mammalian lamin B location at the spindle nuclear envelope (matrix) has already been well documented, as well as the Ran GTP/Run GDP-dependent transport during mitosis [114,115]. This report confirms that during meiosis 1, a similar mechanism is employed. In meiosis II, lamin C is also dispersed through the cytoplasm during metaphase, while lamin Dm forms several to many aggregates within the cytoplasm. Mislocation of lamin Dm affects the meiotic spindle phenotype and chromosomes distribution. Additionally, the localization of lamin C depends on the presence of lamin Dm, as silencing *lamin Dm* expression led to a significant reduction in lamin C levels. In contrast, silencing the *lamin C* gene did not impact the nuclear levels of lamin Dm or its distribution [103]. Overall, the study suggests that, during meiosis, similarly to mitosis, lamin Dm is critical for proper microtubule network function, and its location depends on interaction with Nup107. Lamin C seems to not be involved in interactions with the microtubule network. Since lamin C seems to also be dispensable for embryonic and larval mitosis, it is safe to assume that lamin Dm, similarly to vertebrate B-type lamins, plays a critical role in important nuclear functions.

Lamins play critical roles in building karyoskeletal structures and, together with the LINC complex and several integral membrane proteins, link the nuclear lamina and chromatin to the rest of the cell’s skeletal structures and extracellular matrix. Therefore, lamins play an important role in nuclear positioning, movement, and migration. Regarding lamin Dm, this protein is involved in receptor cell movement [116] and border cell migration [110]. The specific roles of lamin C and its interactors, including membrane proteins and LINC-associated proteins, are discussed in further sections.

In vitro studies have demonstrated that, similarly to mammalian B-type lamins, lamin Dm interacts with chromatin and histones, can assemble into lamina-like structures in in vitro nuclear assembly assays, and associates with the inner nuclear membrane and its integral membrane proteins, including LINC complex proteins, thus taking part in cellular structural and mechanical systems in a manner similar to human B-type lamins [52,95,117,118,119].

Lamin Dm, in association with topoisomerase II, plays a vital role in nuclear matrix and scaffold structures. Upon heat-shock induction, the expression levels of both lamin Dm and Top2 increase in the nuclear matrix structure. Notably, the nuclear matrix and karyoskeletal framework disassemble when subjected to RNase treatment. This phenomenon is observed not only in multicellular animals, but also in plants [74,120,121,122,123].

Studies in vivo in mammalian and *Drosophila melanogaster* cells have shown a close association between lamins and chromatin fibers [124]. In vitro studies have revealed specific binding between lamins and chromatin fragments or interphase chromatin [8,125,126], mitotic chromosomes [127,128], and condensed chromatin assembled in vitro [129]. There have been no doubts that lamin Dm protein properties and functions are similar to vertebrate B-type lamins, especially those of mammalian lamin B1 and B2. Moreover, lamin Dm plays several additional functions in *Drosophila* that, in mammals, are attributed to lamin A. Most notably, it regulates structural and functional chromatin domains, both directly and indirectly, via a network of interactions with almost all nuclear and NE components including NPCs, LINC complexes, chromatin, and chromatin components. Therefore, lamin Dm has been considered a B-type lamin with some extra functions that have been attributed to mammalian lamin A, especially in respect to regulation of chromatin structure and gene expression regulation [84,130]. The properties of *Drosophila* lamin C, which functions as an A-type lamin, will be discussed in the following sections.

## 5. *Drosophila* Lamin C Structure and Function

The gene encoding the second intermediate filament protein in *Drosophila* was cloned in 1993 [131]. Sequence analyses classified the protein as a nuclear intermediate filament, specifically a lamin, based on its structural features, which closely resembled those of mammalian lamin C. Accordingly, the protein was designated as lamin C [39,132]. Lamin C protein expression is developmentally regulated, and protein expression starts predominantly in differentiating cells. Fly lamin C lacks the CaaX farnesylation motif, resembling the mammalian C-type lamin in this way, but its overall expression pattern follows the general trend characteristic for mammalian A- and C-type lamins. Knockout of lamin C is lethal, while general overexpression of lamin C seems to be lethal in a stage-specific manner, suggesting important, tissue-specific functions [133,134]. The lethality observed upon lamin C knockout and overexpression typically occurs at the pupation stage (Table 1) highlighting the importance of maintaining appropriate lamin C levels at this stage and supporting its tissue-specific roles.

*Drosophila* lamin C consists of 621 amino acid residues, while human lamin A and lamin C contain 664 and 572 residues, respectively. The sequence identity between *Drosophila* lamin C and human A-type lamins is approximately 37.5%. Both *Drosophila* lamin C and human lamins A and C are present in the NE [39]. Fruit fly lamin C has a molecular weight of 69,860 Da, whereas human lamin A has 74,139 Da and human lamin C has 65,135 Da. Vertebrate lamins can form heterodimers in vitro [10] while, in fly models, lamin Dm and lamin C form independent networks in vivo, as observed in mammalian cells [47,100].

**Figure 2:**
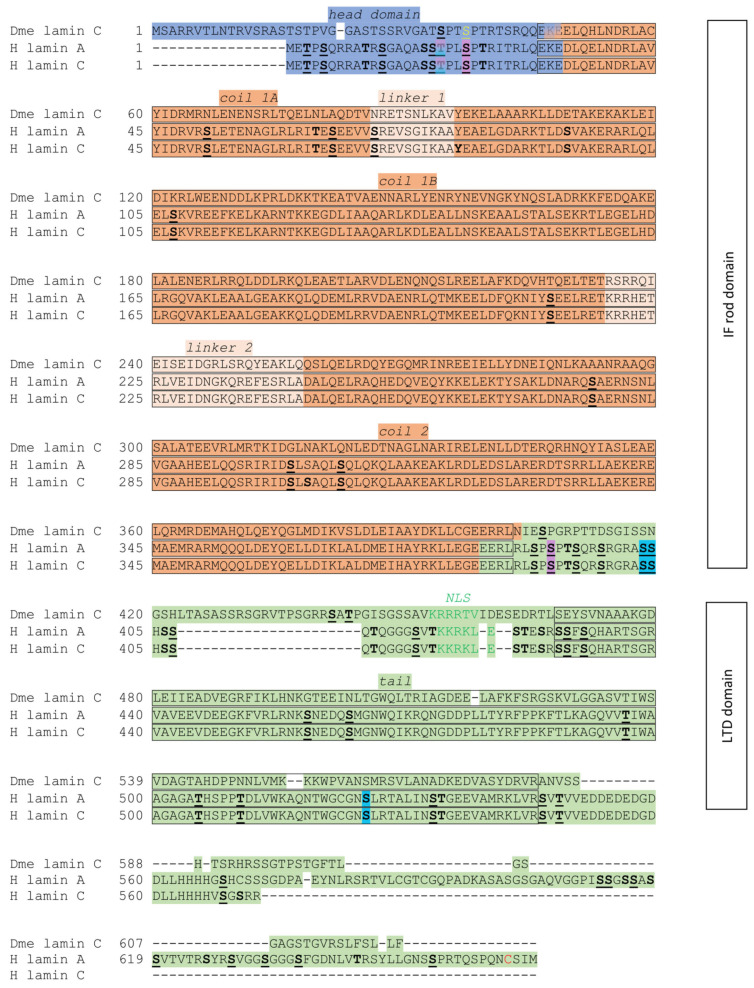
Multiple sequence alignment for A-type lamins from *Drosophila melanogaster* (Dme) and *Homo sapiens* (H), with a particular focus on the lamin structural elements and their similarity between sequences. Alignment prepared using Clustal Omega (clostalo 1.2.4 online version; https://www.ebi.ac.uk/Tools/msa/clustalo/ (accessed on 19 March 2022) with the default algorithm). Sequences, structural regions, and motifs were downloaded from the UniProt and iPTMnet phosphorylation motif databases (validated on 15 February 2021). Description of color marks and symbols used in alignments: **S, T,** and **Y**—bolded and underlined amino acids depict the phosphorylation site according to UniProt and iPTMnet databases. **S, T,** and **Y**—bolded amino acids depict the phosphorylation sites according to iPTMnet with additional data sources: phospho.ELM, PhosphoSitePlus, and Human Protein Reference Database (HPRD). S—confirmed experimentally, in the case that the UniProt database lacks information about this particular site; S (yellow and underlined)—experimentally confirmed but not annotated in the UniProt database; red C—S-farnesyl cysteine site. Phosphorylation sites: cyclin-dependent kinase 1 phosphorylation site (Cdk1)—purple; protein kinase C alpha type phosphorylation site (PKCα)—cyan. Changes in lamin isoforms from interphase to M-phase isoforms are connected with phosphorylation and dephosphorylation events [52,76,78,80,81,137,138]. Lamina disassembly and nuclear envelope disassembly are correlated with lamin phosphorylation caused by Cdk1 protein kinase and protein kinase C [78,139,140,141]. Notably, the N-terminal Cdk1 phosphorylation site at S37 plays an important role in lamin C polymerization and solubility. The impact of the S37L mutation on *Drosophila* larval muscle structure further underscores its functional significance (see Table 2) [52,142].

The level of *Drosophila* lamin C expression, as with human A-type lamins, depends on the developmental stage. Northern blot analysis of embryos allowed for the detection of lamin C transcripts in 12 h embryos, while the highest level of transcripts was observed in 19 to 22 h embryos. In situ hybridization revealed the initial expression of lamin C in oenocytes of stage 12 embryos. Expression in stage 13 embryos was detected in the foregut, hindgut, oenocytes, and dorsal longitudinal trunks. The highest level of lamin C expression was observed in larvae, after which it decreased in pupae and adults [132]. The *Drosophila* lamin C head domain has over a dozen residues, more than the human lamin C and lamin A head domains. This domain is very important for the head-to-tail polymerization of lamins [90]. *Drosophila* lamin C, human lamin A, and lamin C contain a cyclin-dependent kinase 1 phosphorylation site (Cdk1) and a protein kinase C alpha phosphorylation site (PKCα). All amino acids that indicate phosphorylation sites are shown in Figure 2. This lamin C also has two copies of the SR/RS dipeptide motif, which is specific for SRPKs. There is also a motif that is similar to the motif S/TRAS/T in B-type lamins; in the fruit fly, it is localized in the head domain, whereas, in humans, it is in the tail domain. The rod domain has three coiled-coil domains, which are separated by linker regions. This domain is essential for the formation of coiled-coil dimers [41,135]. The C-terminal tail domain has a Cdk1 site, Ig-fold, and nuclear localization sequence (NLS) [60]. The tail domain is shorter in the fruit fly lamin C than in human lamin A, but longer than that of human lamin C (see Figure 2) [136].

It has been shown that a lack of A-type lamins does not interfere with viability of cultured cells [143]. Studies focused on the knockdown of lamin C in *Drosophila melanogaster* revealed larval lethality in pre-pupal stages (Table 1). These results suggest that lamin C is necessary for the proper development of fruit flies [133,144]. Other studies implementing lamin C knockout or knockdown in muscles demonstrated aberrant nuclei shape, muscle structure, and disturbed lamin filaments [133,145,146,147,148].

Further evidence of the tissue-specific expression and function of lamin C was obtained via lamin C-specific expression in the CNS using the GAL4/UAS system. No significant differences were observed between the test and control samples. These results indicate that the role of lamin C is tissue-specific [149], as ectopic expression of lamin C appears to be both stage- and tissue-specific, predominantly resulting in lethality at the pre-pupa stage [134]. These data suggest a similar role of fly lamin C as mammalian A-type lamins in terms of differentiation [150,151].

Interesting data have been obtained through lamin C overexpression: characteristic phenotypes included melanotic tumors, lethality in larva, and features generally similar to null mutants of *dcp-1* (a gene coding for apoptotic caspases) (Table 1) [134,147]. Lamins are substrates for caspases, and the expression of mutated lamins that are resistant to caspase activity inhibited cell apoptosis [152]. When lamin C was overexpressed by the *ptc*-GAL4 driver in A/P boundary cells of *Drosophila melanogaster*, DNA degradation and apoptosis were detected, together with highly condensed chromatin and decreased levels of lamin Dm and lamin C. Apoptosis was also confirmed via the ability to inhibit cell death by P35 (baculovirus caspase inhibitor) [149].

The polytene nuclei (from the salivary gland) and diploid nuclei (from imaginal disks) of *Drosophila LaminC^EX187^* (flies with a deletion in the first exon of the *lamC* gene that are protein-null) third-instar larvae were analyzed to investigate whether the absence of lamin C caused any alterations in the localization of nuclear envelope proteins [147]. No changes were observed in localization of Bocksbeutel (LEM-domain protein) and HP1a staining in null mutants [147]. These results might suggest that lamin C is not essential for the proper localization of heterochromatin and Bocksbeutel in fruit flies, at least in tested tissues. In mammalian cells (tongue epithelium, skeletal muscle, ventricular cardiacmuscle) a lack of A-type lamin resulted in the presence of emerin (LEM-domain protein) clusters, a reduced level of emerin staining, and changes in the localization of that protein from the nucleus to cytoplasm, in comparison to wild-type mice [147,153]. As lamin C does not interact with chromatin and DNA, both in vitro [52] and in vivo [93], while lamin Dm does, the lack of effect on heterochromatin in fly polytene salivary glands nuclei is not surprising, especially considering that fly lamin Dm lacks the farnesylation motif associated with polytene chromosomes, while lamin C and membranes with INM proteins retain wild-type staining [95,96]. It is possible that B- and A-type lamins exhibit distinct interactomes at the nuclear lamina and the INM in association with NPCs, both in *Drosophila* and in mammalian cells [100,154]. However, one study assessing the expression of lamin C RNAi under the control of *ptc*-GAL4 drivers in the salivary glands of third-instar larvae reported a lower level of HP1a, its diffused staining, and decondensed chromatin together with elongated nuclei [149]. A reduction in HP1a protein was also visible in peripodial cells under *Ubx*-GAL4 lamin C overexpression [149]. These results might suggest that lamin C is responsible for the proper distribution of HP1a and HP1a protein-dependent LAD domains in some cell nuclei, or that its overexpression may lead to such a phenomenon in certain cell nuclei. This is consistent with studies in mammalian cells; however, earlier studies in *Drosophila* did not confirm these results, highlighting the need for further research [147,149,153]. Alternative explanation may come from the mouse system and mammalian lamin C. Knockdown of lamin C in MEFs resulted in post-mitotic disturbances in the proper assembly of chromatin LADs. The authors suggested that at normal lamin C level, protein assists in proper LAD formation by polymerization around centrally located, decondensing chromatin till early G1 due to the delayed protein dephosphorylation events [155]. The question remains whether such a mechanism is universal between cell types in mammals and species in particular. Considering that this effect in *Drosophila* was detected in polytenic nuclei previously undergoing rounds of endoreplication, and general differences in histone markers in *Drosophila* lamin Dm LADs, this needs to be verified before considering this a mechanism explaining *Drosophila* study.

**Table 2:** Summary of phenotypic effects associated with various *lamin C* gene mutations in *Drosophila melanogaster*. The table compiles data from experimental studies describing tissue-specific and developmental stage-specific phenotypes. The severity of phenotypic outcomes and associated molecular signatures varies depending on the specific mutation and tissue context.

Reference	Fly Mutation	Tissue/Organ/Stage	Phenotype
[133]	Schulze et al., 2005: 1. wt; 2. G00158; 3. R401K; and 4. ΔN	Salivary glands Epithelial cells	1. Normal nuclear rim staining; 2. O-ring aggregates; 3. strong O-ring phenotype lethality; 4. lamin C/lamin Dm aggregates, prepupal lethality.
[147]	Schulze et al., 2009: 1. N210K; 2. R401K; 3. K493W; 4. W557S; 5. L567P; and 6. ΔN and ΔC	Larval salivary gland nuclei	1 and 2. O-ring lamin aggregates, chromatin defects, viable; 3. normal, viable; 4. chromatin defects, fully lethal for Mef2; 5. granules at NE, viable; 6. diffused internal lamins and chromatin, lethal with some configurations.
[145]	Dialynas et al., 2010: 1. wt; 2. LamC; and 3. ΔN mutant;	Larval muscles	3. ΔN: muscle nuclei defects, lethality, disrupted NE components, cytoskeleton abnormalities, endocrine disruption.
[51]	Dialynas et al., 2012: 1. wt; 2. G489V; 3. N496I; 4. V528P; and 5. M553R	Larval body wall muscles	2. Larval movement defects, extranuclear lamin C granules, NPC, Klaroid, and gp210 mislocalization, disrupted lamin Dm; 3. mild larval phenotype; 4. larval movement defect, lamin C granules, strong cytoplasmic Klaroid and NPC mislocalization, altered lamin Dm pattern; 5. larval movement defect, lamin C granules, NPC and Klaroid mislocalization, viability depends on used promoter.
[44]	Dialynas, 2015: 1. wt; 2. G489V; 3. N496I; 4. V528P; and 5. M553R	Larval body wall muscles	1. Normal distribution of lamin C; similar nuclear strain as in mutants (except ΔN); no accumulation of redox markers; baseline expression of stress-related genes. 2. Upregulation of redox stress markers; nuclear accumulation; activation of Nrf2/Keap1 pathway; 21 genes affected similarly as in ΔN mutant. 4. Activation of redox stress pathway: increased CncC, p62 and Keap1 signal; moderate overlap in transcriptional profile with ΔN and G489V. 5. elevated redox stress markers; transcriptional profile overlaps partially with G489V/ΔN; 21 common genes affected both in ΔN and G489V mutants.
[156]	Chandran et al., 2019: 1. A177P; 2. R205W; 3. G489V; and 4. V528P	Adult indirect flight muscles	Fln-GAL4 driver: held-up wings in 2, 3, and 4; shorter sarcomeres in 2 and 3; disrupted Z-disks and M-lines in 2 and 3. DJ694-Gal4: mild phenotype; lamin/NPC aggregates; flightless phenotype. Rescue via AMPKα, dPGC-1, Thor, or S6K knockout.
[157]	Bhide et al., 2018: R205W, G489V	Semi intact hearts	Myofibril disorganization, lobulated nuclei, cytoplasmic lamin C and Otefin aggregates, elevated lamin C and Ref(2)P levels, more cytoplasmic foci, G489V milder than R205W, age-dependent phenotype worsening, reduced lifespan, enlarged lipid droplets, higher triglycerides, nuclear CncC accumulation. Atg1 overexpression reduces aggregates and improves heart and lifespan; CncC knockdown improves heart but not fat or lifespan; best rescue with combined Atg1 overexpression and CncC knockdown; Atg1 inhibition worsens phenotypes.
[142]	Shaw et al., 2022: 1. S37L; 2. ΔK47; 3. L74R; 4. R205W; 5. R237P; 6. G489V; 7. K521Q; and 8. R564P	Larval body wall muscles	ΔN lamin C: fully viable; 1, 2, 4, 5, and 7: lethal; 3: ~15% viable; 6 and 8: reduced survival; 1, 6, 7, and 8: cytoplasmic granules; 4: lamin C blebs and chromatin/actin defects; 8: granular lamin C at chromatin edge, NPCs at rim and in cytoplasm; NPCs: nuclear in wt and control, reduced in 6 and 7; increased nuclear strain in 2, 3, and 4; increased displacement and microtubule defects in 2 and 7; RNAi against Koi and MSP300 has no effect on nuclear strain.
[158]	Hinz et al., 2021: 1. R264Q; 2. R264W; and 3. R564P	Larval and adult IF muscles	1 and 2: sterile in larval muscle, viable in IFM with age-related wing defects, stronger in females; 3: reduces larval motility; wt: nuclear lamin C with granules, normal NPCs and lamin Dm, thickened microtubules; 1: intranuclear lamin C ovoids, irregular lamin Dm, NPC aggregates, disorganized microtubules; 2: fragmented NE lamin C, lamin Dm in granular NE. protrusions, NPC granules on DNA+/– areas, disrupted microtubules.
[146]	Walker et al., 2023: K521W and R564P	Larval muscles, fat body	1 and 2: sterile; 2: enlarged larval muscles (esp. segment 8), reduced motility, ~50% shorter lifespan in cardiac muscle; wt: lamin C at NE with granules, Otefin in nuclear granules, TMEM43 at NE; 1: lamin C in large NE protrusions, DAPI excluded, Otefin at NE, TMEM43 in protrusions and NE; 2: lamin C c:ytoplasmic near nuclei, Otefin at NE and cytoplasm (no colocalization), TMEM43 mostly cytoplasmic; 2 in fat body: cytoplasmic lamin C, weak NE signal.

In lamin C null mutants (*LamC^LC58^*), larval short stop protein (referred to as shot, a cytoskeletal protein that belongs to the spectraplakin family) was detected at an abnormal location in tendon nuclei [144]. The fragmented distribution of that protein was visible around the nuclear envelope, replacing the continuous layer. In contrast, lamin Dm displayed no apparent abnormalities. Shot protein was more diffused in tendon cells with altered nuclei and incorrectly localized lamin Dm (Table 2; Supplemental Table S1). The disintegration of shot structures and the consequent lack of integrity in the lamin Dm layer suggest that lamin C maintains cytoskeletal structures in a shot-dependent manner, facilitating the strength and proper functioning of tendon cells. The expression of lamin C with *shot*-GAL4 drivers in null mutants *LamC^LC58^* allowed 90% of larvae to develop into pupae. It also rescued more than 80% of head-eversion defects and around 60% of pupae developed into the adult form; however, more than 20% of them had some flying- and walking-related difficulties. Moreover, shot expression was not observed in muscle body cells, and these results might suggest that lamin C is necessary for cells/nuclei that express shot [144].

Overexpression of lamin C in the wing disk using the *Ubx*-GAL4 driver revealed that lamin C and lamin Dm aggregate at the nuclear periphery. Additionally, the expression of UAS-*lamC*-UTR^RNAi^ and UAS-*lamC*-tail^RNAi^ with the ap-GAL4 driver indicated a lower amount of lamin Dm in nuclei. This suggests that both a lack of and overexpression of lamin C disrupt the nuclear lamina (see also Table 2 and Supplemental Table S1 for comparison of lamin C overexpression). Overexpression with the driver how24B-GAL4 caused defects related to the longitudinal muscles’ formation in larvae and lethality at the first stage, while the driver *pnr*-GAL4 in adults disrupted the organization of indirect dorsal flight muscles [149]. Alternative/additional mechanisms may act through the short stop protein. Since lamin C is responsible for proper localization of the short stop protein, important for MTJ formation [144], this may explain the lethality of the phenotype. Additionally, a lack and overexpression of lamin C induces reorganization of the NL/NE, lamin Dm level, and distribution. This may lead to the rearrangement of lamin Dm TADs in chromatin and relocate the chromatin protein itself. For example, the activity of chromatin-associated Winged-Eye protein, which is necessary for imaginal disk trans-determination, depends on the proper level and location of lamin Dm [159]. This indicates that not only can the decreased level of lamin C induce phenotypes in a tissue-selective manner, but it also confirms previous data that tissue-selective overexpression of wt lamin C can induce a phenotype that can also be lethal. This is important, especially when we consider the phenotype developed in experiments with lamin overexpression, especially in lamin C mutants resembling lamin A laminopathy mutants.

## 6. Muscle-Related Laminopathies Based on Fly Lamin C

It is commonly believed that *Drosophila* lamin C has the same general properties and plays similar functions to mammalian A-type lamins, especially in terms of its structural and functional roles in cell nuclear functions, as well as in the development of muscles [133,153]. This view has also been supported by numerous in vivo knockout and knockdown studies, as well as many in vitro studies (as discussed in the previous section). As concluded in the previous section, fly lamin C does not fully recapitulate all of the mammalian lamin A features and functions.

As the fly system possesses only two lamin genes and two lamin proteins (lamin B-type and lamin A-type)*, Drosophila melanogaster* can be considered an appropriate, convenient, and relatively easy model for studying human laminopathies in vivo. An additional advantage of the fly model is its simpler genome/transcriptome/proteome; for example, many myogenic factors and regulatory pathway proteins are represented by a single gene/protein. As a typical example, the fly Mef2 transcription factor is represented by a single gene, while, in the mammalian genome, there are four equivalent transcription factors (i.e., MEF2A, B, C, D) [160]. An additional advantage of *Drosophila* studies is the availability of a powerful genetic system for the manipulation of gene expression (i.e., GAL4) and comprehensive fly GAL4 strain libraries [161]. When we consider *Drosophila* as a model for laminopathies, the unquestioned major contributor to such studies is Lori Wallrath lab, both alone and with collaborators [146]. The very first published observations contributing to the connection of fly lamin C phenotypes, especially in larval body wall muscles [133], with mammalian lamin A phenotypes [153], opened the subject of regarding fly lamin C as a model for the development of rare disorders such as laminopathies. Lamin null larval muscle nuclei displayed abnormal, typically elongated morphology, with aberrant, mostly dispersed staining for lamin Dm. While cytoplasmic actin appeared normal, polymerized actin structures were observed within the nuclei. These intranuclear structures are partially the “nuclear actin bars” seen when actin export is inhibited [109]. This might be the tissue-specific role of lamin C, since in S2 cells, the actin bar structures were lamin Dm-, exportin 6-, and Nup98-dependent. Later studies demonstrated that the substitution mutants R401K (human: R386K), K493W (human: R453W), W557S (human: W520S), and L567P (L530P), which were developed to reflect human mutations that cause autosomal dominant Emery–Dreifuss muscular dystrophy (AD-EDMD), as well as N210K (human: N195K), causing dilated cardiomyopathy (DCM), when expressed in larval body wall muscles, indeed induced a laminopathy-like phenotype when expressed in larval body wall muscles [147] (for details on phenotype and mutant type, see Table 2 and Supplemental Table S1). Mutant lamin C was visible as aggregates in nuclei of imaginal disks, salivary glands, epithelial tissue, and the gut at the second stage of larval development.

Exploration of the salivary gland of third-instar larvae revealed a reduced amount of lamin C at the nuclear periphery. Furthermore, 50–100% of all nuclei showed lamin C aggregated in O-ring structures, and lamin C aggregates frequently occurred with lamin Dm aggregates [133]. The same results were obtained after designing the rod-domain mutation N210K, corresponding to the human N195K mutation [147]. A similar nuclear phenotype was observed in human A-type lamin rod-domain mutations [133,147,162]; however, the flies remained viable without any visible defects [147].

The *Drosophila* mutants K493W and W557S correspond to the human lamin A mutations R453W and W520S, respectively. Contrary to previous results (i.e., in mutants N210K and R401K), nuclei of the salivary gland from third stage larvae presented an accumulation of lamin C staining in the nuclear periphery. Moreover, in L567P mutants (reflecting human L530P), lamin C not only accumulated at the nuclear rim but also formed discrete intranuclear foci resembling those observed in mammalian cells expressing the L530P variant [147,163]. Mutants that had peripheral localization of lamin C were lethal [147]. After expressing the substitution mutants using the GAL4-UAS system driven by Act 5C, ey, elav, Mef2, How24B, and T80 promoters, most of them were viable. Only the W557S mutant was lethal, but only with the Mef2 promoter, and reduced viability was observed after expression with Act 5C, T80, and how24B promoters [147]. These results, together with the discovery of internally polymerized actin inside nuclei, may implicate an important role of lamin C in muscular development both at the muscle body and myo-tendon junctions (MTJs). Truncation of the N-terminal domain caused Autosomal Dominant EDMD (AD-EDMD)—a neurogenic variant—and two different constructs of the N-terminal-truncated lamin C. In the first construct, eight amino acids were deleted from the rod domain in addition to the lack of the N-terminal domain [133]. Expression of this modified protein caused aggregates of O-ring structures in cell nuclei, a reduction in lamin C staining in the nuclear periphery, and the death of flies at the pre-pupal developmental stages. Aggregates in nuclei were caused by the deletion of amino acids from the rod domain, while early lethality was caused by truncation of the N-terminal domain.

The second construct was deprived only of amino acids, which were included in the N-terminal domain [147]. In contrast to the first construct, this one revealed an accumulation of lamin C at the nuclear periphery. Expression of this truncated protein using the GAL4-UAS system under the control of Act 5C, How24B, T80, and Mef2 promoters caused lethality [145,147]. The importance of the head domain of lamin C may result from several factors. The most important Cdk1 (“mitotic”) site in lamin C is located at S37, together with several other important kinase sites, including the SRASTSTP motif with PKC and PKA sites [60]. Pseudophosphorylation of this sole site (S37E) made lamin C in S2 cells soluble, similar to T435E (C-terminal Cdk1 site) on lamin Dm, and blocked its association with decondensed chromatin in the *Xenopus* nuclear assembly system [52]. In the fly model, the lamin Dm N-terminus and the beginning of the rod domain play an important part in the maintenance of proper conformation, similar to human lamin A; in this regard, S25 phosphorylation seems to be important [83]. Further research was conducted by the same group, with a focus on muscle promoters. Expression via larval muscle promoters such as Act 5C, how24, C57, and Mef2 caused lethality or reduced viability. There were no changes in viability when mutated lamin C was expressed by promoters in adult muscle [145].

To detect abnormal nuclear phenotypes, larval body wall muscles of third instar larvae were analyzed. Staining of the muscles with lamin C antibodies revealed the aggregation of lamin C in nuclei. Nuclei that expressed the mutated protein were misshapen, elongated, and presented highly condensed chromatin; furthermore, 9% of nuclei showed nuclear pore complex aggregation, slightly more than 10% of nuclei showed Otefin (Emerin ortholog) and Klaroid (*Drosophila* SUN-domain protein) aggregation, and the actin–tubulin cytoskeletal network was also disorganized. This suggests that not only lamin Dm but also lamin C plays a role in microtubule anchorage, via LINC complex proteins. This also suggests the important role of lamin C in muscle proper development but not in adult muscle function. Please note that early muscle promoters such as Mef2 and how24 are active in precursor nuclei/mioblasts for main body nuclei, MTJ nuclei, and imaginal disks. The latter’s proper functions are necessary for proper adult phenotypes, e.g., survival/lethality/aging speed (see Table 2 and Supplemental Table S1). Further studies have shown that lethality was the result of nuclear and cytoplasmic defects in body wall muscles, and flies that reached adulthood presented leg defects [145]. Expression of W557S and N-terminal-truncated lamin C using the GAL4-UAS system revealed important roles of the Ig-fold and N-terminal domains in muscle [147]. The substitution of mutant W557S takes place in Ig-fold, which probably participates in binding to DNA and likely takes part in lamin assembly. It is important to note that C-terminal truncation does not cause lethality, and the mutation W557S is only expressed in muscle. This is likely due to the interaction of that domain with muscle proteins, which is necessary for proper muscle development [147]. It has been suggested that abnormal protein expression of lamin C in larval body wall muscles induces dystrophic features with less regular fibers, as revealed on cross-sections stained for dystrophin and aberrant lamin C staining at the nuclear envelope, but with normally laterally located cell nuclei in muscle fibers [51]. Interestingly, there is a correlation between the extent of the phenotype and the amount of lamin C relocated to aggregates, especially cytoplasmic, as well as lamin Dm staining and LINC complex component staining being disturbed (Table 2; Supplemental Table S1).

Further studies aimed at finding mechanisms associated with muscle-related laminopathies in *Drosophila* larval body wall muscles as a model involved lamin C ΔN, G489V, V528P, and M553R mutants mimicking mammalian lamin A mutants (G449V, L489P, and W514R, respectively) [44]. Mutant lamin C M553R demonstrated the strongest phenotype, with the highest level of mutant protein and the highest level of protein in cytoplasmic aggregates. The second, in terms of phenotype and cytoplasmic aggregates, was the lamin C mutant V528P, while G489V demonstrated a weaker phenotype in both criteria and was only a little stronger than lamin C ΔN. Surprisingly, the lamin C mutant N496I matched the control phenotype. For analysis of the levels of oxidized glutathione (GSSG)—an oxidative stress marker triggering associated stress response through the Nrf2/Keap-1 pathway, reduced glutathione (GSH), and NADPH—only lamin C mutants G489V and ΔN were considered. GSH and NADPH are effector molecules of enzymatic activities—with respect to glutathione S transferases, HO-1 heme oxygenase-1 (HO1), and NQ01 NAD(P)H:quinone dehydrogenase 1 (NQO1)—of genes activated by the Nrf2 transcription factor. No statistical differences in the level of GSSG in body wall muscles were detected when compared to the lamin C control, although ΔN showed an increasing trend. Meanwhile, the GSH content was much higher in both mutants compared to the control, although lower in G489V compared to ΔN, and NADPH was increased in both mutants by about five-fold compared to the control. Differential gene expression analyses of the mutants G489V and ΔN revealed 21 common genes that were affected in both mutants. Only two of them were expected for potential Nrf2 targets: *GstD9* (glutathione S reductase D9) and *Cyp4p2* (heme binding, iron binding, oxidoreductase activity) [157,164,165].

Interestingly, redox equilibrium and heme/iron metabolism seem to play a role in the development of the Duchenne muscular dystrophy phenotype. This might suggest at least a partly common pathogenesis mechanism between EDMDs and DMD [166]. Immunofluorescence analyses of body wall muscles allowed for the detection of increased levels of CncC protein (dNrf2) in dispersed loci, as well as cytoplasmic increases in G449V, L489P, and W514R. The original appropriate human lamin A mutants in biopsies from appropriate patients also induced increased levels of Nrf2, presenting similar changes as those induced by lamin C mutants. Lamin C mutants and human lamin A mutants in biopsies induced cytoplasmic staining for p62 protein, targeting the Keap1 protein for autophagic degradation. The authors suggested that the lamin A mutants induce phenotype directly in patients, while lamin C mutants induce phenotype in a manner mediated through reductive stress [44].

Other studies have reported reductive stress induced by Nrf2 pathway activation due to sulforaphane-impaired myogenic differentiation [167] and that Nrf2 regulated viability, proliferation, stress resistance, and differentiation in a murine myoblast cell line [168]. Others analyzed oxidative stress and inflammation as important factors in DMD, suggesting that inflammation might be the primary inducer, through L-6, NOX2, Nrf2, and its target pathways [169]. Some authors have suggested the prospect of DMD therapy based on the targeting of Nrf2 [170]. The question is whether inflammation is a primary or co-primary inducer of the phenotype in *Drosophila* and human models of dystrophic laminopathies (and DMD) or, instead, a secondary inducer. Alternatively, cytoplasmic lamin aggregates recruit a significant fraction of p62 proteins and direct them for autophagy, which, in turn, increases the level of Nrf2 protein due to the constant degradation of Keap1 and autosomal degradation involved in the processing of lamin aggregates, consequently inducing reductive stress. This interpretation might be supported by the beneficial effect of Nrf2 knockdown in decreasing the dystrophic phenotype, both in *Drosophila* and mammals [171,172,173]. An important discovery regarding the role of the Nrf2 pathway [157] is that increasing autophagy and blocking Nrf2 may suppress age-dependent, laminopathy-induced cardiac dysfunction and shorten lifespan. While this study was also performed in a *Drosophila* model, the overexpression of lamin C mutant proteins was performed in the heart. The authors proposed a model of action for Nrf2, in which elevated Keap1, associated with the p62 complex containing lamin C aggregates, stimulates TOR, which, in turn, inhibits the processing of p62–lamin C mutant aggregates via autophagy [157]. To make the problem more complicated, in response to xenobiotic stress, the Keap1 pathway in *Drosophila* is postulated as being affected by interactions with lamin Dm as well [174], possibly affecting developmental transcription in this way [175].

It should be pointed out that some interpretations of the mechanisms in phenotype development may be further complicated by the experimental systems that have been used. Most GAL4-mediated expressions of mutant lamin C have been performed with a hsp70 core promoter, which makes these expression systems responsive to elevated temperatures and associated heat-shock induction. Typically, induction protocols involve 45 min exposure to 37 °C, followed by a 2 h recovery period before preparation of tissues. This treatment induces a heat-shock response in Kc and S2 cells, as well as in embryos, larvae, and flies [176,177,178]. Induced proteins and their transcripts vary in half-life from minutes (HSF transcript) to hours. For hsp70, protein half-life varies depending on tissue, but for culture cells, it is at least several hours approximately [83].

A slightly modified *Drosophila* adult muscle model based on the indirect flight muscle (IFM) was utilized to look deeper into the mechanism of phenotype development in flies overexpressing lamin C mutants [156]. In this study, A177P, R205W, G489V, and V528P mutants, together with wild-type lamin C, were overexpressed using IFM-specific promoters (i.e., Act88F-Gal4, DJ694, and Fln-Gal4). The Fln promoter was active during maturation, while DJ694-Gal4 was active only in fully matured muscles, and the Act88F promoter was activated earlier in development. Homozygous mutant protein expression (Fln-GAL4) affected the flight index (ability to fly), Z and M lines, and sarcomere length. The flight index was only mildly affected in the A177P mutant and more strongly affected in the R205W mutant. In both of them, the inability to fly increased with time from 3 days to 3 weeks. G488V and V528P mutants were unable to fly. When DJ-694-Gal4 was used to overexpress mutant proteins, all mutants were only mildly but significantly affected, with the phenotype increasing between 3 days and 3 weeks of age (Table 2; Supplemental Table S1). Differences between the three drivers may derive from the difference in onset of expression after enclosure and/or strength of the promoters. The mutants induced the presence of lamin C and NUPs in cytoplasmic aggregates, as well as misshapen mitochondria. The fly mutants differed in the extent of misshapen nuclei and the number, size, distribution, and distance of aggregates to cell nuclei. Wild-type lamin C and mutant lamin protein levels differed by about two-fold, which can be explained by the slower turnover of mutant lamin due to its accumulation in aggregates, as has been demonstrated for progerin (lamin A mutant inducing HGPS) in mice. Furthermore, the level of the Ref(2)P protein (p62 in mammals)—a marker of aggresome complexes—was increased [179].

When wild-type lamin C and mutant G489V flies were further modified by Gal4-mediated overexpression and downregulation of S6K and Thor (4E-BP1), restoration of a wild-type-similar phenotype was observed with S6K downregulation and Thor overexpression. The other (opposite) variant had no effect on increase or decrease with respect to the initial G489V mutant phenotype. Downregulation of S6K and overexpression of Thor also reduced the number of lamin C aggregates, as well as the wing phenotype mitochondrial distribution (Table 2; Supplemental Table S1).

Based on the results, the authors proposed a new model of muscle laminopathy development in *Drosophila* adult muscles, which is independent of the Keap1–Nrf2 reductive/oxidative stress pathway. The proposed model assumes that the rescue of wild-type phenotype depends on Thor (4E-BP1) overexpression (inhibitor of non-stress translation) or FOXO overexpression, leading to an increase in Thor level, which should lead to translation inhibition. S6 ribosomal kinase (S6K) downregulation also leads to translation inhibition and rescues the wild-type phenotype, similarly to AMPKα and PGC1α overexpression. The AMPKα pathway rescues the proper levels of PGC1α and FOXO. Therefore, this model assumes that translation inhibition (of non-heat-shock-associated transcripts) rescues the wild-type adult muscle phenotype in *Drosophila*. The authors suggested overexpression of AMPKα as a potential target of therapy, together with S6K, Thor, and FOXO [156]. It is interesting to consider the mechanisms leading to restoration of the wild-type phenotype when non-stress-associated translations are being blocked. One might speculate that reductive stress [44] or oxidative stress, when mTOR kinase is activated, may induce the Keap1–Nrf2 pathway, thus speeding up autophagy and removing lamin C/NPC aggregates through aggresomes. The latter might be facilitated by the shutdown of non-stress-related mRNA translation, also for the aggregated mutant lamins. This hypothesis might be backed up by HSF protein activation and the initiation of a general stress response. Please note that the preparation procedure itself (45 min at 37 Centigrade) may generate induction of HSF via heat shock. Then, the expressed heat-shock proteins, together with Nrf2-target transcripts, might assist lamin aggregates and help solubilize them, further increasing mutant lamin degradation [44].

The classical larval body wall muscles have been used for the expression of lamin C mutants, with mutations mimicking lamin A laminopathy mutations, in order to assess the effect of mutant proteins on nucleo-cytoskeletal coupling [142]. The following lamin C mutants mimicking lamin A laminopathies mutants were developed (lamin C/lamin A, respectively): ΔN/ΔN, S37L/S22L (N-terminal Cdk1 mitotic sites), ΔK47/ΔK32, K74R/L59R, R205W/R190W, R237P/H222P, G489V/G449V, K521Q/R482Q, and R564P/R525P (Table 2; Supplemental Table S1). The S37L mutant demonstrated dispersed punctate cytoplasmic and nuclear staining, as one might expect from lamin C protein lacking N-terminal mitotic Cdk1 sites (as well as PKC and PKA sites), being unable to solubilize. No muscle phenotype (wild-type phalloidin pattern and proper distribution of nuclei detected) was detected for this mutant. In the R205W mutant, an aberrant distribution of nuclei in muscles was observed, particularly misshapen nuclei with nuclear location of mutant lamins at a higher level and in some granules. The mutant protein R235P localized mostly to the cytoplasm around irregular nuclei, with aberrant distribution in muscles. The mutant proteins G489V, K521Q, and R564P were predominantly cytoplasmic. In all mutants except for ΔK47, a significantly lower amount of staining for nuclear NPCs was observed (about 50%). In two lamin C mutants (ΔK47 and K521Q) measurements detected reduced nucleoskeletal–cytoskeletal coupling, while in three mutants (ΔN, L74R, and R205W), increased nuclear strain was detected. Two mutants (ΔK47 and K521Q) were observed to exhibit disruption in the formation of a nuclear cage, with disorganized tubulin networks instead of forming parallel arrays according to muscle length. RNAi-mediated knockdown of the *Drosophila* LINC complex components koi, MSP300, and klar disrupted perinuclear microtubules. It is intriguing to hypothesize that centrosomes in ΔK47 and K521Q mutant cells may also be displaced from the NE. In fibroblasts from patients with EDMD1 (emerin null), the distance of centrosomes from the NE was increased when compared to control fibroblasts [180,181,182]. Based on the above data on phenotypes generated by wt lamin C and its mutants’ expression, it is possible to conclude that they do not provide clear evidence on the lamin C interactors. Despite the fact that wt lamin C forms granules at the NE and in cytoplasm, which can also recruit lamin Dm [51,147], both NE proteins, Otefin and TMEM43, do not follow the distribution of lamin C fully (Otefin in granules; TMEM43 at the NE) (Table 2; Supplemental Table S1). This is also true for at least several lamin C mutants (e.g., K521W or R564P), which suggests that lamin C is not the only interactor for Otefin and TMEM43.

A combination of *Drosophila* larval body wall muscles and adult indirect flight muscles (IFMs) was used to analyze newly generated lamin C mutants, bearing equivalent mammalian lamin A mutations discovered in dystrophy-related laminopathies. For a deeper study, two lamin C mutants were selected: R264Q and R264W. These mimic the lamin A coil2A mutants R249Q and R249W, which are associated with AD-EDMD and lamin-related CMD (L-CMD), respectively. They were overexpressed in larvae via the C57 driver and in IFMs using the Act88F driver [158].

Overexpression in larval muscles of wild-type lamin C resulted in punctate distribution with standard nuclear shape and NPC distribution; meanwhile, both mutants induced similarly lobulated nuclei, but only R264Q induced redistribution of NPCs. In IFMs, both mutants induced held-up wings in up to over 90% of males (R264W) and up to 50% of females. For R264Q, this was up to 50% in males and up to about 25% in females. Interestingly, mutant R264Q induced a more dramatic phenotype—displaced, fused-together NPCs, higher expression level, and abnormal chromatin distribution—but differing from the other mutant, it was much weaker in inducing the phenotype in IFMs. The authors, using in silico-predicted models, discussed the structure of mutant lamin A and possible interaction pockets within the Ig-fold domain [158]. The next report was a continuation of the above-mentioned studies, this time using *Drosophila* larval body wall muscles, fat body, and cardiac muscle cells [146], considering two lamin C mutants, R521W and R564P, which mimic lamin A myopathy mutants in the Ig-fold domain (R482W and R527P, respectively). A similar lamin C mutation (R521Q) and the same R564P had already been analyzed earlier [142]. Overexpression in body wall muscles was driven by C57, while cardiac targeting was achieved using the dHand4.2 promotor specific to cardioblasts, pericardial nephrocytes, and hematopoietic progenitors. For larval fat body lamin C mutant overexpression, two specific drivers were used (P{Lsp2-Gal4.H}3 stock #6357 and P{r4-Gal4}3, stock #33832; Bloomington Stock Center). In body wall muscles, the K521W mutant induced an interesting nuclear phenotype with a single, half-spherical protrusion strongly stained with lamin C Abs and the vast majority of DNA staining out of protrusion. This nuclear phenotype resembled the phenotype of nurse cells in oocytes with Jill-1 kinase knockdown, which may suggest that this mutant might have induced an aberrant and persistent connection to the microtubule network through association with LINC complex proteins [183]. The R564P mutant was mostly cytoplasmic, similar to the previous study, with DNA/chromatin distributed laterally. Overexpressed wild-type lamin C was distributed more or less typically, but with multiple granule-like spots. Under wild-type lamin C overexpression, Otefin was distributed only inside cell nuclei with punctate staining, while Transmembrane Protein 43 (TMEM43, also known as Luma) was located exclusively at the NE/NL with occasional small, circle-like external protrusions.

It may suggest that removal of lamin C, together with Otefin, from the NL to the nuclear interior disconnects TMEM43 from NL/NL/chromatin contacts, resulting in the formation of NE protrusions with TMEM43. This may indicate lamin C–Otefin interactions and suggest lamin C as an interactor of TMEM43. Since lamin C also interacts with microtubules, through the LINC complex, these lamin C interactions strongly resemble mammalian lamin A interactions. Taking this into account, combined with previous experiments on Otefin and TMEM43 staining, we may safely conclude that both lamin Dm and lamin C and other NE interactors are involved in the anchorage of Otefin and TMEM43 at the NE. Since lamin Dm has been reported to interact with Otefin and YA [184], lamin Dm is also an interactor. Similarly complex interactions occur with NPC proteins. Mouse monoclonal antibodies MAb414 recognize many “soluble” NPC proteins with FG-repeat domains; therefore, the heterogenous, not comparable distribution of staining for different lamin C mutants strongly suggests either multi-protein dependence of location or different interaction partners for particular sets of nucleoporins (e.g., lamin Dm but not lamin C interaction/colocalization for Nup107 and Nup98) [103,114].

TMEM43 interacts with emerin, lamin A, and SUN2, which are LINC complex component proteins. In particular, mutations in its gene result in EDMD7 [185] in *H. sapiens*. In the K521W mutant, Otefin localized mostly to lobulations at the NE/NL, while TMEM43 was mainly localized in single protrusion as punctate staining with a minor fraction of TMEM43 at the NE/NL in the nucleus (Table 2; Supplemental Table S1). This phenotype again suggests the role of this lamin C mutant in forming a permanent complex with Otefin, TMEM, LINC complex components, and the cytoplasmic microtubule network, thus stabilizing protrusions. In the R564P mutant, Otefin localized mostly to the NE/NL, with insignificant punctate cytoplasmic and nuclear staining. It is interesting to note the percentage of nuclei with single or many lobulations in the K521W mutant and the differences in Otefin and TMEM43 distributions (punctate or at the NE/NL) between strains. The fat body phenotype was similar to the muscle phenotype for mutants. Mostly single protrusions for K521W and mostly cytoplasmic staining for lamin C mutants were detected, with cells in mutants being smaller compared to lamin C-overexpressing cells. As expected, both mutants were practically not viable. dHand4.2-driven expression of wild-type lamin C and mutant expression resulted in shortened lifespan: by about 50% for the R564P mutant and the same longevity as with wild-type lamin C overexpression for K521W [146] (Table 2; Supplemental Table S1).

## 7. Progeria HGPS and Other Rare Disorder Models Based on Lamin C

Based on the data discussed above, it is obvious that the *Drosophila* model is an excellent model for deep in vivo studies of the mechanisms underlying phenotype development in patients. Surprisingly, some earlier studies in *Drosophila* focused on the specific features of the CaaX (farnesylation) motif in proteins, which may also contribute to understanding the mechanism underlying HGPS and the features of fibroblasts observed in patients. In the cells of HGPS patients, especially at the higher passages and/or lower proliferation rate, the most prominent and easiest to spot phenotype is characterized by shape aberration and multi-lobulation of cell nuclei. A key question is whether this is the cause of the disease at the metabolic or transcription level or the effect of simple accumulation of additional lamin A with a retained CaaX motif at the nuclear envelope. The extra copies of NE proteins may accumulate over time and if cell divisions are rare, then the protein accumulates [104,112,186,187,188,189,190].

Reports on farnesylated lamins, especially B-type lamins, accumulating in slowly dividing or post-mitotic cells are in favor of the latter hypothesis. Indeed, a high level of overexpression of lamins affects the shape and phenotype of the nuclei in *Drosophila* and mammalian cells. Additionally, in *Drosophila*, overexpression of farnesylated lamin Dm, lamin C, Kugelkern (farnesylated CaaX motif), transmembrane proteins, and protein domains with the CaaX or GFP-CaaX motif induce lobulations, membrane stacks, and “annulate lamellae” [104,105]. Moreover, overexpressed farnezylated lamin Dm and Kugelkern promote aging-like phenotypes in *Drosophila* [189], affect nuclear shape in intestine and enterocytes [111], affect the liposome membrane in vitro [190], and regulate nuclear size and shape during embryo development [112]. Since FTI inhibitors work well in alleviating the nuclear phenotype in fly models of HGPS [188], *Drosophila* can be a good model for HGPS studies [191], and reports from fly models may significantly contribute to the understanding of HGPS development and the role of a large quantity of additional farnezylated proteins (progerin) at the nuclear envelope.

Indeed, the hypothesis of a direct correlation between the level of progerin at the NE and the extent of aberrant nuclei phenotypes, concluded from *Drosophila* models, can be confirmed by mammalian models. In patient-derived fibroblasts, nuclear abnormalities have been shown to become more pronounced with increasing passage number and, consequently, with progerin accumulation [186,192]. This was also shown to correlate with progerin expression level and reverse upon its downregulation [193,194]. Moreover, recent papers have demonstrated that in newly isolated fibroblasts from HGPS patients, only 7.2% of cells display misshapen nuclei, while in MEFs, it was 13% in heterozygous and 24% in homozygous cells. In HeLa and NHDF cells after transient transfection with GFP-progerin, typically leading to substantial overexpression, 29% of cells had misshaped nuclei. Importantly, mammalian model data nicely correlate with *Drosophila* data showing that the amount, ratio, and composition between NL/NE proteins, either farnezylated or with transmembrane domains, strongly affect the aberrant nuclei phenotype frequency and extent. This confirmation comes from a recent paper demonstrating the effect of manipulations of lamin A levels or emerin levels in a cellular HGPS model [195]. See also the effect of overexpression of fly lamins (also withouth farnezylation motif) and human lamins in fly and HeLa cells, as well emerin deletion mutants in human cells and their different effect on location and nuclei aberrations [52,196].

Another direct support for this hypothesis comes from studies in mammalian models, where progerin can be produced in large quantities in cardiovascular tissues (aorta, heart) and fat, and may be especially long-lived, similar to B-type lamins [197]. Recent data from various animal models of HGPS, with tissue-selective progerin expression, strongly indicate that a simple high level of progerin in a particular tissue is not sufficient to induce the HGPS phenotype in such tissue [198,199]. Indeed, B-type lamins are long-lived proteins in the nervous system, lasting up to years [200,201]. Please note that neurons and muscle cells do not proliferate and, so, might accumulate a high quantity of lamins, which can affect tissue function, especially when proteins aggregate. In the *Drosophila* larval body wall muscle model, mutated lamin C designed to bear equivalent lamin A mutations caused a dystrophic phenotype, but generally, only aggregating mutants and with a high amount of lamin C (and associated proteins) induced a strong phenotype with reductive stress, aberrant protein turnover, and Keap1–Nrf2 pathway induction [202]. This was observed independently of whether the mutants localized to the cell nucleus or to the cytoplasm, either in proximity to or at a greater distance from the nucleus (see above) [146]. In mouse models of HGPS, when RNA therapy was tested, progerin persisted in the heart after up to 5 months of treatment [203]. A recent study has demonstrated that lamin A/C, and especially progerin, are more resistant to solubilization in the cardiovascular tissues and, therefore, are more resistant to degradation/autophagy. Furthermore, progerin is longer-lived compared to lamin A and induces aberrant “turnover of hundreds of abundant proteins in progeroid tissues” [179]. It appears that *Drosophila* and mouse models provide similar hints as to the pathological mechanisms of HGPS and, perhaps, dystrophy-type laminopathies.

The *Drosophila* model system has been widely used as a general model of muscle development [43,144,204,205,206], as well as for specific issues associated with the formation of neuromuscular and neurotendonous junctions [207]. The development of the nervous system and neuronal circuits has also been studied in *Drosophila* [19]. Furthermore, human neurodegenerative disorders have been studied using the *Drosophila* model system [20], and, when assessing several neurodegenerative human disorders, collectively called Tauopathies, via this model, lamin Dm dysfunction and/or cytoskeletal–nucleoskeletal coupling were postulated as mediating factors in the development of neurodegeneration [208]. *Drosophila* models have been used as a genetic resource of mutants, in order to potentially better understand the mechanisms underlying human genetic disorders [209]. Of particular use was the generation of a collection of X chromosome mutants. For example, taking advantage of the opportunity provided by the created mutants, it was possible to identify Ankle2 (Ankyrin repeats LEM-domain protein2) null mutants as being responsible for the disruption of asymmetric cell division in *Drosophila* neuroblasts, thus causing microcephaly. Similarly, in humans, ANKLE2 has been linked to hereditary microcephaly [210].

*Drosophila melanogaster* lamin C mutants resembling human lamin A dystrophic laminopathy mutations present nuclear defects and muscle abnormalities. This makes the fruit fly a relevant model for studying laminopathies, which may be helpful in better understanding the functions of A-type lamins. There are still many aspects remaining to be explained and understood, and research focused on the effects of lamina C on various functions or mechanisms occurring in cells should be further conducted. The fact that there is still no effective commercial therapy for laminopathies is an important reason why significant attention should be paid to these topics.

## 8. Summary of Discussion of Laminopathies and Other Rare Disorders Model Based on Lamins

Genomic analyses suggest that *Drosophila* lamin Dm is the ancestral gene for both lamins and the broader intermediate filament protein family [4,39]. The emergence of lamin C in *Drosophila* likely resulted from a duplication event of the lamin Dm gene, explaining the higher similarity between the two fly lamins compared to lamins from other species. Importantly, *Drosophila* encodes only nuclear intermediate filament proteins (type V), with no cytoplasmic IF protein/gene detected [37,84]. Despite this, *Drosophila* and vertebrate lamins share conserved gene expression patterns, functional domains, phosphorylation and farnesylation sites, and structural elements. Lamin Dm fulfills criteria for a B-type lamin and offers a simplified model, as flies possess a single gene and protein versus multiple B-type lamins variants in vertebrates. Lamin C is structurally similar to A-type lamins but lacks farnesylation and does not bind DNA/chromatin in vivo, suggesting similarity to human lamin C. However, its capacity to induce dystrophic phenotypes aligns it functionally with lamin A [38,60,107].

Phosphorylation of lamin Dm has been extensively studied in *Drosophila*. The nascent Dm_0_ polypeptide is phosphorylated into Dm_1_, and later (for example under stress conditions) into Dm_2_, likely at the S25 residue [71,72]. Similarly to mammalian B-type lamins and lamin A, lamin Dm is phosphorylated at N- and C-terminal “mitotic” Cdk1 sites (lamin Dm_mit_). Notably, phosphorylation at the C-terminal site alone induces disassembly, while single N-terminal phosphorylation (e.g., S45) does not. Additional residues (S42, S48, S50, R48, and R84) modulate head–coil 1A interactions, regulated by proteins like 14-3-3 and Pin1. The C-terminal Cdk1 site also has two phosphoacceptors: T432 and T435 [60].

Only lamin Dm binds DNA/RNA in vivo and tethers chromatin to the nuclear lamina. During mitosis, it associates with mitotic membranes, being partly dispersed and partly forming the spindle nuclear envelope, similarly to mammalian B-type lamins. *Drosophila* lamin Dm and C form distinct networks. Non-farnesylated lamin Dm targets the protein to the surface of chromatin, at least in some tissues, whereas lamin C remains attached to the NE/NL together with other NL proteins [52]. *Drosophila* lamin C appears to share several features with mammalian lamin A and some with lamin C. Knockout of lamin C is lethal in a stage-specific manner, as is its exogenous, tissue-specific expression. Many lamin C mutants that mimic *LMNA* mutations induce muscular dystrophy-like phenotypes when expressed in flies. These phenotypes are associated with activation of xenobiotic stress responses, the CncC (dNrf2)/Keap1 pathway, and deregulation of over 20 genes. Similar mechanisms have been observed in Duchenne muscular dystrophy (DMD), including aberrant Nrf2 signaling and reductive stress. The severity of the phenotype strongly correlates with the aggregation of mutant lamin C, p62, and nuclear proteins, alongside dNrf2 upregulation and inflammation. Notably, Nrf2 knockdown improves outcomes in both *Drosophila* and mammalian models, highlighting its central role in dystrophic pathologies [44,145].

Professor Lori Wallrath’s group reported that *Drosophila* muscle laminopathies can be rescued independently of Keap1–Nrf2 signaling through overexpression of Thor, FOXO, AMPKα, or PGC1α. This underscores the importance of translation inhibition and mitochondrial regulation in phenotype suppression [159,160,161,172]. These findings suggest that blocking non-stress-related translation may promote autophagic clearance of lamin aggregates, potentially aided by HSF1-mediated induction of heat-shock proteins (HSPs) [159].

This hypothesis aligns with experimental observations on the effect of mutants’ overexpression where heat stress response is likely induced (e.g., 37 °C for 45 min). Thus, both inhibition of non-stress-related translation and heat stress-induced HSP expression may synergistically suppress translation of aberrant lamin C mutants and enhance solubilization and degradation of aggregated lamin C and associated proteins. Furthermore, the aforementioned signaling pathways (Thor/4E-BP, FOXO, etc.) may delay or prevent recovery from heat shock, particularly the resumption of non-stress-related translation.

Interestingly, the K521W lamin C mutant induces characteristic half-spherical nuclear protrusions that are strongly stained with lamin C antibodies, with most DNA displaced from the protrusions. This phenotype resembles nuclear defects observed in nurse cells with Jill-1 kinase knockdown and suggests abnormal, possibly persistent coupling of lamin C to the cytoskeleton via the LINC complex [178]. Based on mutant phenotypes, lamin C likely interacts with Otefin, TMEM43, and LINC complexes, and its loss leads to nuclear deformation and cytoskeletal disorganization [145]. *Drosophila* lamin C mutants thus recapitulate key features of laminopathies, confirming the fly as a relevant model organism. Given the lack of effective therapies, *Drosophila* offers a valuable platform for investigating pathogenic mechanisms and therapeutic strategies.

The fly model may also provide insights into Hutchinson–Gilford progeria syndrome (HGPS), where nuclear lobulation and accumulation of farnesylated lamins (e.g., progerin) occur in non-dividing cells [180]. These lobulations, prominently visible in fibroblasts from HGPS patients—especially in late passages—are widely considered a hallmark of the disease. Their disappearance is often used as a marker of treatment efficacy. In *Drosophila*, overexpression of farnesylated lamins or CaaX-tagged proteins (e.g., GFP-CaaX) induces similar phenotypes, including nuclear lobulations and membrane structures [102,103]. Comparable phenotypes can also be observed in *Drosophila* and mammalian cells overexpressing lamin Dm, lamin B, or prelamin A. Please see page 31 for a deeper discussion. In mammals, progerin is particularly long-lived in post-mitotic tissues and resists degradation, contributing to persistent pathology despite RNAi-based therapeutic interventions [173,185]. Moreover, in various animal models of HGPS, tissues contributing to the pathological phenotype often show progerin levels similar to surrounding tissues, yet no lobulation phenotype has been observed. These findings support the hypothesis that impaired autophagy and proteasomal turnover of lamins, combined with mechanical/cellular stress and the ratio between progerin and other NL/NE proteins (including lamin A)—rather than overexpression alone—drive disease progression. Strikingly, in both *Drosophila* and mouse models, lamin C or progerin aggregation, oxidative/reductive stress, and disrupted clearance mechanisms emerge as common pathological themes in dystrophic laminopathies, DMD, and progeroid syndromes [145,172,184].

*Drosophila* has been extensively used to study muscle development [144], neuromuscular and myotendinous junctions [189], and nervous system architecture [19,21]. It has also contributed to modeling neurodegenerative diseases, including tauopathies, where lamin Dm and nucleoskeletal defects are implicated [190].

Key nuclear proteins—such as LEM-domain proteins, LINC complex components, nuclear pore complex (NPC) proteins, and LBR—have orthologs in both humans and flies. Although the fly LEM-domain protein family is smaller than its human counterpart, many fly proteins remain functionally uncharacterized. Additionally, other fly proteins may perform similar roles. Therefore, the *Drosophila* model is highly valuable for studying conserved mechanisms relevant to both flies and humans [211].

## 9. Lamin Dm and Lamin C Interaction Networks

**Figure 3:**
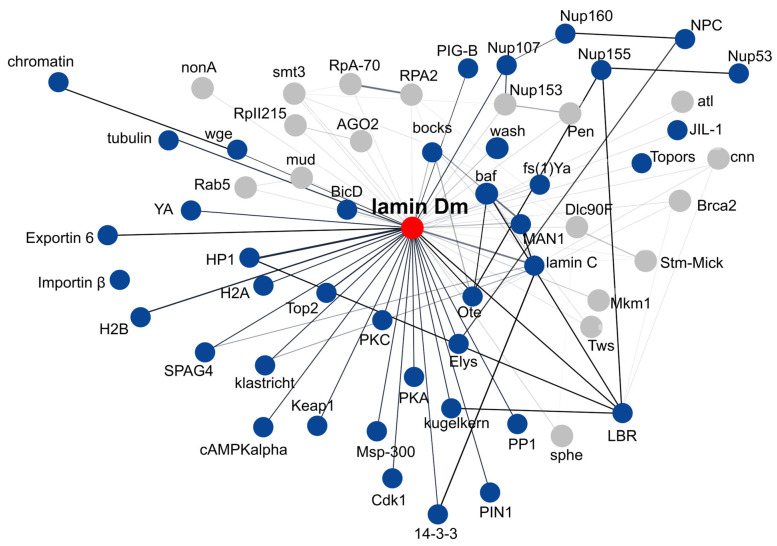
Interaction network for lamin Dm. The protein–protein interaction network was constructed using the STRING DB (v12.0) and then visualized and further supplemented with additional nodes in Cytoscape v3.10.3. Additional significant interactions were added to the network manually based on the literature analysis. The network in STRING was created on the basis of physical interactions, as evidenced by experimental results, with a low confidence threshold of 0.150. The scheme presents two categories of protein–protein interaction: blue coloration is assigned to nodes that have been experimentally confirmed to be associated with their respective partners; and gray nodes represent other reports suggesting interaction.

**Figure 4:**
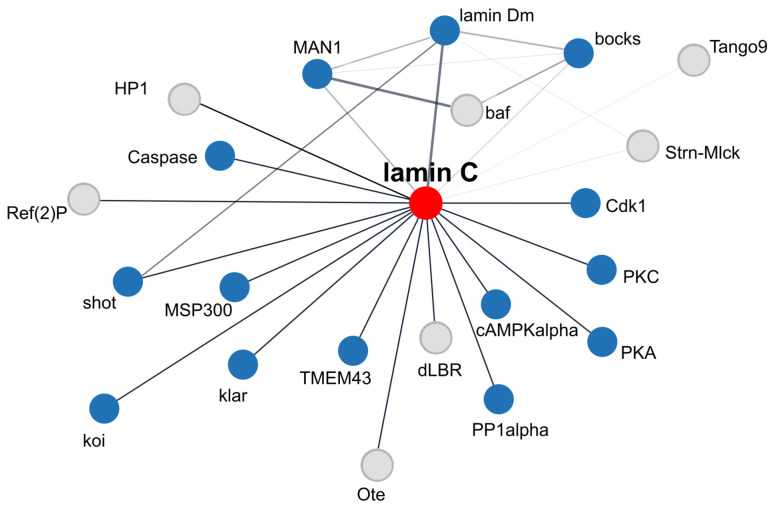
Interaction network for lamin C. The rules for creating interactions are identical to those for lamin Dm (see Figure 3).

Fly lamin Dm and lamin C have been thought to be similar in structure, properties, and functions to B-type mammalian lamin (but not lamin B3) and a mix of lamin A and lamin C, respectively. Therefore, their networks of interactions should reflect their counterparts. It is well known that the knowledge of mammalian lamin interactomes far exceeds the knowledge of fly ones. Taking this into account, it is intriguing how similar, based on current data, both animal interactomes are. Bioinformatic analyses with the use of String software (v12.0) and our personal validation and modifications allowed us to create a map of interactomes for lamin Dm and lamin C (Figure 3 and Figure 4, respectively). For visualization of manually edited interactome network, we used Cytoscape software (v3.10.3). For the lamin Dm interactome, we mostly focused on demonstrating only the nearest protein partner from longer chains of interactions. This was due to the need for clarity and simplicity of the presented lamin Dm network. Only in the case of several interactors, which, we believe, are important for this review, we extended the chain of interactors. For example, we had to present a complex network of interactions connecting lamin Dm, Nup107-Nup160, NPCs, Elys, dLBR, Nup155, Nup53, Otefin, BAF, and the LEM-domain protein network (Figure 3). For the lamin C network, mostly because of limited experimental data, we could present only the first interacting proteins (Figure 4).

Please note that we have not taken into account any potential interactions based on delocalization of potential interaction partners by lamin levels or mutations unless there has been proof that this might suggest direct interaction not related to common clustering in aggregates. The lamin Dm interactome map (Figure 3) demonstrates all known and suggested interactions with NE proteins, such as LINC complex proteins [212,213], dLBR, Otefin, YA, BAF, bocksbeutel, and dMAN1 [214,215,216,217,218]—for review, see [68,211]; at least some of the NPCs and soluble nucleoporins, e.g., Nup107, Nup98, and Elys [103,219,220,221,222,223]; chromatin components [52]—for review, see [224]; Topo1 and Topors1 [225]; Topo2 [83]; histones H2A and H2B [119,226]; Bicaudal D [134]; and many other nuclear or NE proteins, such as PIG-B [227,228]. Please note the variety of protein kinases and phosphatases marked as interactors, as well as proteins involved in modulation of phosphoproteins such as PIN1 [229]; (see also Milbradt et al., 2016 [230]) and 14-3-3 protein [231].

There have been additional problems associated with direct association of particular interactions of lamin Dm and lamin C with potentially common interactors such as LINC complex proteins, some LEM-domain proteins, LBR, or HP1a. Some of the published data may suggest lamin Dm or lamin C, both, or neither as interactors. Additionally, interactions may vary depending on a particular tissue or stage of development since not all potential interactors (e.g., lamin C itself) are expressed at a certain stage/tissue as lamin Dm is. Therefore, based on the strength of evidence for the interactions and expression patterns of the proteins in question, we have decided to associate LBR indirectly with both lamins and directly with the Elys interactome, while MAN1 is associated with lamin Dm. There has been a problem in properly associating HP1a protein (and interactome)—some studies suggest interaction/colocalization at the NE with lamin Dm, but not with non-farnezylated lamin Dm (e.g., in polytenic nuclei), when HP1a associates with chromatin while HP1a stays at the NE/NL. Similarly, lamin Dm knockdown in embryonic Kc cells (no lamin C) does not relocate HP1a from the NE, which suggests NE recruitment and loading through Elys and the interacting Nups network via connection with chromatin at the end of mitosis, together with LBR or independently (Figure 3). The latter data correlate with the existence of separate lamin Dm (LADs; not enriched in H3K9me2) and HP1a chromatin domains in Kc cells. In other tissues (central brain, glia, Elav-positive neurons, fat body) chromatin domains of lamin Dm (LADs), HP1a and PcG overlaps [28].

A similar, complex network of interaction exists when one considers lamin Dm, LEM-domain proteins, BAF, and LINC protein complex interplay. Both lamins interact with bocksbeutel in Y2H while other studies confirm only Otefin and bocksbeutel as interactors for lamin Dm, together with other LEM-D proteins: dMAN1 and CG3167 [216]. All LEM-D proteins interact with BAF, so they should have at least some overlapping functions through this interaction. Deletion mutant studies of single proteins give no phenotype, but any combination of double mutants gives strong phenotypes in flies, which suggest separate interactomes, except for BAF interactions. It looks like Otefin is linked to TGFβ signaling while bocksbeutel associates with Smads regulation [217]. Otefin and bocksbeutel also play important but different roles in interaction with LINC complex proteins, or at least with Klarsicht and tubulin networks outside nuclei, in the positioning of cell nuclei in developing muscle. Loss of bocksbeutel reduced nuclear Klarsicht, while loss of Otefin increased the transcription and nuclear Klarsicht, both affecting nuclear positioning. Surprisingly, double mutants restored the normal localization of nuclei [212]. This suggests the opposite association of complexes with tubulin motors. Indeed, bocksbeutel was detected to be responsible for Klarsicht–tubulin links between nuclei, while another protein, enconsin, was responsible for nuclei transport on tubulin [213]. Otefin also plays an essential role in connecting nucleoporin networks to the NL/NE. Otefin’s connection with the nucleoporin–chromatin network takes place through several interactions (Figure 3): the BAF interactome [215], Nup155 and Nup53, Nup93 and Elys to NPCs, and through the lamin Dm network [232].

Otefin is also essential, together with BAF protein, in germline stem cell (GSC) development, regulating cell cycle checkpoints. In GSCs, Otefin is required for the activity of Dpp/BMP signaling pathways, probably via BAF protein [215], direct interaction with Medea/SMAD4 at the bam silencer element, and regulating asymmetric division [233,234,235]. The dKeap1 protein is an intriguing interactor for lamin Dm and lamin C (Figure 3 and Figure 4). We have already discussed the role of the Keap1-dNrf2 (CncN) pathway in response to xenobiotic stress and oxidoreductive stress in the context of *Drosophila* lamin C laminopathy mutations above. The Keap1-Nrf2 pathway has also been involved in the targeting and activation of developmental genes in *Drosophila*, such as adipogenic genes [175], and in the activation of metamorphosis by promoting ecdysone synthesis and response genes [236]. Its interaction with lamin Dm provokes discussion on the potential alternative or at least additional molecular mechanisms for *Drosophila* lamin C laminopathy mutant phenotypes. Keap1 and lamin Dm overexpression in *Drosophila* results in lethality at larval stage 2. Keap1 interacts with lamin Dm, relocates lamin to the nuclear interior, and spreads heterochromatin, marked by H3K9me2, which in turn affects LAD distribution and gene expression, including Nrf2-dependent genes [174]. This may suggest that lamin C laminopathy mutants affecting lamin Dm distribution, likely inducing higher levels of Nrf2 and also affecting gene expression via this mechanism. Similar mechanisms of enhancing or modulating lamin C mutants’ phenotypes may be activated via nucleoporin/NPC component mislocalization. In *Drosophila*, many nucleoporins play a critical role in chromatin reassembly, proper mitotic spindle assembly, proper location of NL/NE proteins, and proper tethering chromatin domains, including binding to active genes (see Figure 3 and Figure 4) [222,237,238,239]. Lamin C laminopathy mutant-induced relocation/aggregation of nucleoporins/NPCs may induce remodeling of chromatin domains and affect gene expression patterns [240,241]. In this context, several candidate nucleoporins are evident, including Nup107-160 complex, Nup155, Elys, and associated proteins such as dLBR, HP1a, or Otefin [232] (see Figure 3).

Wash is a *Drosophila* protein of the WAS (Wiskott–Aldrich syndrome) protein family, playing a role in cytoskeletal organization, signal transduction, and membrane trafficking. Cytoplasmic WAS proteins are effectors for Rho family GTPases, polymerizing branched actin via the Arp2/3 complex [242,243,244]. *Drosophila* wash plays a role in global nuclear organization and the redistribution of chromatin domains with repression markers. Mutants’ nuclei exhibit wrinkled morphology. *Drosophila* wash is distributed in cytoplasm and cell nuclei, where it interacts with lamin Dm [245].

Lamin C interactome data are much scarcer, especially if we do not consider data on the redistribution of potential partner proteins due to knockdown or mutants’ overexpression. There has been an additional problem associated with the direct association of particular interactions of lamin C with potentially common interactors such as LINC complex proteins, LEM-domain proteins, and LBR. Some of the published data may suggest lamin Dm, lamin C, or both as interactors (see discussion in the upper section). Therefore, the lamin C interactome consists of kinases and phosphatases, modifying the polymerization and interactions of lamin C, LINC complex proteins, and some LEM-D proteins [211]. We think that further studies on *Drosophila* lamins, especially lamin C, will discover many new interactors. Anyway, both lamins create independent networks for NL and NE protein interactions and, by “immobilizing” protein complexes in gaps of the created “fishing net”, maintain proper distribution of proteins (in domains? [246]), protein complexes, or entire NPCs.

## 10. Conclusions

In conclusion, *D. melanogaster* is proving to be an extremely valuable model for the study of lamins and lamin-associated proteins. The simplicity of genetic manipulation in this organism enables fast and easy investigations. Furthermore, the high degree of evolutionary conservation in the structure of cell nuclear proteins means that results obtained in the fruit fly are often transferable to higher organisms, including *H. sapiens*. Consequently, the fruit fly offers an invaluable tool for understanding the pathogenetic mechanisms of laminopathy and, consequently, in the search for potential therapies.

## Figures and Tables

**Table 1:** Summary of phenotypic effects associated with genetic manipulation or loss-of-function mutations in the *lamin Dm* gene in *Drosophila melanogaster*. The table compiles data from experimental studies describing tissue-specific and developmental stage-specific phenotypes. The severity of phenotypic outcomes and associated cellular effects varies depending on the nature of genetic manipulation and tissue context.

Reference	Type of Manipulation or Strain	Tissue, Organ, Stage	Phenotype
[84]	Lenz-Böhme et al., 1997 *Lam^P^* (P-element insertion mutant in lamin Dm)	Larvae, pupae, adults	Developmental delay; impaired locomotor responses; minority (5–10%) reached adulthood; adults displayed sterility; abnormal gonadal development; defective oogenesis; malformed nuclei; nuclear pore complexes (NPCs) clustered and mislocalized; lamin Dm protein remained detectable in tissues despite phenotypic abnormalities.
[106]	Guillemin et al., 2001 misg^sz18^ (loss-of-function allele of lamin Dm)	Germline, embryos, larval stages	Disturbed dorsal–ventral egg polarity in oocyte (*gurken* transcripts and Gurken protein mislocalization); reduced levels of lamin Dm in oocyte and nurse cells; normal level in epidermal cells/
[43]	Osouda et al., 2005 1. *Lam^14^/Df*; 2. *misg^sz18^/Df*; 3. *Lam^14^/CyO*; 4. *Lam^P^*; 5. *Lam^14^/Lam^P^*; 6. *Lam^9^*; 7. *Lam^14^*	CNS, imaginal disks, gonads, digestive tract, pupae, third-instar larvae	1. Lamin Dm absent in the CNS at L3. Overall, ~76% survival to the late pupal stage; ~1% reached adulthood. 2. No lamin Dm expression in CNS at L3; late pupal survival 77%; adult emergence ~3%. 3. Normal lamin Dm expression, fertility, and 100% adult emergence. 4. Lamin Dm expressed in larval brain and imaginal disks. In total, 89% survived to late pupal stage; 65% reached adulthood. All adults were sterile. 5. Lamin Dm absent in CNS and imaginal disks at L3; 72% survived to pupation; 45% became adults; and 45% of the adults were sterile. 6. Complete absence of lamin Dm in L3 CNS and imaginal disks. Overall, 59% pupal survival; no adults emerged. 7. Lamin Dm detectable in the CNS at L2 but undetectable at L3. Defective gonad formation, delayed CNS development, proliferative abnormalities in the larval midgut. EcRB1 levels reduced. Approximately 63% reached the pupal stage; no adult flies emerged.
[108]	Chen et al., 2014 Gal4-UAS knockdown Cg-Gal4/+; tub-Gal80ts/Lam RNAi	Fat body, midgut	Age-related lamin B depletion in the fat body: transcriptional dysregulation, upregulation of over 100 immune-related genes, systemic inflammation observed, IMD pathway hyperactivation, progressive midgut hyperplasia, indicating a loss of heterochromatin-mediated gene silencing, and impaired tissue homeostasis.
[109]	Dopie, 2015 RNAi-mediated knockdown of lamin Dm, Nup98, and exportin 6	Cultured S2 cells, male meiotic cells	Lamin Dm depletion in cultured cells: disrupted nuclear actin organization lead to altered localization of actin and dysregulation of cofilin regulators. In the male germline, concurrent knockdown of lamin Dm and Nup107 impaired cytokinesis and mislocalization of nuclear lamins,.
[103]	Hayashi et al., 2016 Gal4-UAS; lamin Dm and Nup107 knockdown	Neural stem cells, larval neuroblasts	Simultaneous depletion of lamin Dm and components of the Nup107–160 complex compromised the integrity of the spindle envelope during mitosis. This disruption led to misalignment of chromosomes, spindle pole defects, and impaired mitotic progression, suggesting a cooperative role of lamin and NPCs in mitotic architecture.

## Data Availability

Authors state that no new experimental data have been created in relation to this publication.

## Supplementary Materials

The following supporting information can be downloaded at: https://www.mdpi.com/article/10.3390/cells14171303/s1, Table S1: Detailed phenotypic characterization of *Drosophila melanogaster* models expressing wild-type and mutant forms of *lamin C*. The table summarizes results from experimental studies, utilizing tissue-specific GAL4-UAS and heat-shock-inducible systems to drive expression in various organs. Phenotypic outcomes vary by mutation, expression level, and tissue-specific context. Sources: Schulze et al. (2005, 2009) [133,147], Dialynas et al. (2010, 2012, 2015) [44,51,145], Chandran et al. (2019) [156], Bhide et al. (2018) [157], Shaw et al. (2022) [142], Hinz et al. (2021) [158], and Walker et al. (2023) [146].

## Abbreviations

The following abbreviations are used in this manuscript:

NE	Nuclear envelope
LINC	Linker of nucleoskeleton and cytoskeleton
EDMD	Emery–Dreifuss muscular dystrophy
ADLD	Autosomal dominant leukodystrophy
CMT	Charcot–Marie–Tooth
HGPS	Hutchinson–Gilford progeria syndrome
APLD	Acquired partial lipodystrophy (Barraquer–Simons syndrome)
LGMD	Limb–girdle muscular dystrophy
PHA	Pelger–Hue’t anomaly
HEM/GRBGD	Greenberg hydrops-ectopic calcification-moth-eaten skeletal dysplasia
BOS	Buschke–Ollendorff syndrome
ECM	Extracellular matrix
INM	Inner nuclear membrane
NL	Nuclear lamina
IF	Intermediate filaments
NLS	Nuclear localization signal/sequence
NPC	Nuclear pore complex
Cdk	Cyclin-Dependent Kinase
PKA	Protein Kinase A
PKC	Protein Kinase C
Gsk	Glycogen Synthase Kinase
ERK	Extracellular Signal-Regulated Kinase
MAPK/APK	Mitogen-Activated Protein Kinase/Activated Protein Kinase
S/MAR	Scaffold/matrix attachment region
cAMP	Cyclic Adenosine Monophosphate
GFP	Green Fluorescent Protein
CNS	Central Nervous System
IMD	Immune deficiency
Top2	Topoisomerase II
SRPK	Serine/Arginine Protein Kinase
LEM	LAP2-Emerin-MAN1 Domain Protein
MEF	Myocyte Enhancer Factor 2
MYF	Myogenic Factor
DMD	Duchenne Muscular Dystrophy
TOR	Target of Rapamycin
Nrf	Nuclear Factor Erythroid
NOX	NADPH Oxidase
IFM	Indirect flight muscle
AMPK	Activated protein kinase
FOXO	Forkhead box O
NPS	Neuropeptide S
HSF	Heat Shock Factor
CMD	Congenital muscular dystrophy
TM	Transmembrane
TMEM	Transmembrane Protein
BAF	Barrier-to-Autointegration Factor
LAP	Lamina-associated polypeptide
MAN	Inner Nuclear Membrane Protein
LEMD1	LEM Domain-Containing 1
EMD	Emerin
ANKL	Ankyrin Repeat and LEM Domain-Containing
BOKS	Barrier-to-Autointegration Factor (BAF)-binding protein of KASH domain
OTE	Otefin
NES	Nuclear Export Signal
MSC	MAN1/Src1p/C-terminal motif
ANK	Ankyrin repeats
RM	RNA Recognition Motif
GIY-YIG	Endonuclease Domain (a conserved DNA-binding motif)
LBR	Lamin B Receptor
HEM	Hydrops-Ectopic calcification-Moth-eaten skeletal dysplasia
LBS	Lamin binding site
CHD	Chromodomain-Helicase-DNA-binding protein
ABS	Acting binding side
AMC	Arthrogryposis multiplex congenital
MCPHRb	MicrocephalyRetinoblastoma
GSSG	Glutathione Disulfide
GSH	Reduced glutathione
wt	Wild type
