# Supplementary material for: Drosophila as a Model for Studying the Roles of Lamins in Normal Tissues and Laminopathies
Source: Cells. 2025 Aug 22;14(17):1303. doi: 10.3390/cells14171303 (PMC12428385; doi:10.3390/cells14171303)
Supplement: Supplementary file 1 [file cells-14-01303-s001.zip › cells-3769987-supplementary.pdf]

Supplementary Table S1. Detailed phenotypic characterization of *Drosophila melanogaster* models expressing wild-type and mutant forms of *lamin C*. The table summarizes results from experimental studies, utilizing tissue-specific GAL4-UAS and heat-shock–inducible systems to drive expression in various organs. Phenotypic outcomes vary by mutation, expression level, and tissue-specific context.. Sources: Schulze et al. (2005, 2009), Dialynas et al. (2010, 2012, 2015), Chandran et al. (2019), Bhide et al. (2018), Shaw et al. (2022), Hinz et al. (2021), Walker et al. (2023).

References	Fly mutation/ human mutation, construct	Tissue/ organ/ stage	Phenotype
[1]	1. wild-type LamC<br>2. G00158 (parental line) LamC-GFP exon trap; also disrupts *ttv*<br>3. R401K LamC mutant (GAL4- and heat-shock-inducible; homologous to human R386K LMNA causing EDMD),<br>4. ΔN-truncation - heat-shock-inducible<br>expressed from vectors pUAST (GAL4 inducible) and pCaSpeR-hs/act (heat-shock inducible);<br><br>Reference: Schulze et al. 2005	Salivary glands Epithelial cells (aggregates also observed in brain, imaginal discs, gut)	**1.** Normal nuclear rim staining<br>**2.** Moderate O-ring aggregates in lamin C and lamin Dm (~40–50% nuclei)<br>**3.** Strong O-ring phenotype in lamin C and lamin Dm (50–100%)<br>**4.** Multiple lamin C/lamin Dm aggregates, prepupal lethality
[2]	GAL4-UAS mediated expression of lamin C point mutants with drivers: hsp70, Mef2, Act5C, how24B, elav, ey. Construct: pCaSpeR-hs/act (heat-shock inducible)<br><br>Mutants:<br>1. N210K/hN195K<br>2. R401K/hR386K<br>3. K493W/hR453W<br>4. W557S/hW520S<br>5. L567P/hL530P<br>6. ΔN and ΔC<br><br>Reference: Schulze et al 2009	Third intar larvae, Salivary gland, nuclei<br><br>High level of overexpression	1. Many O-ring structures with lamin C only, in nuclei; diffused chromatin; viable with all promoters<br>2. Less O-ring structures with lamin C, in nuclei; diffused and grainy chromatin; Viable with all promoters<br>3. Normal; viable with all promoters<br>4. Normal lamins distribution; diffused and lateral chromatin; fully lethal only for Mef2;<br>5. Granules at NE with lamin C and lamin Dm; viable for all promoters.<br>6. ΔN and ΔC – normal with diffused internal lamins and chromatin; ΔN-lethal for Act5C,T80, how24B and Mef2; ΔC-fully viable.
[3]	GAL4-UAS mediated overexpression of wt lamin C and lamin C ΔN mutant<br>Construct: pCaSpeR-hs/act (heat-shock inducible).<br>Selected promoters:<br>Act5C, C57, How24B, Mef2, MHCF3, C380, CCAP<br><br>Reference: Dialynas et al 2010	Larval muscles Muscle and brain specific promoters	Wt lamin C overexpression: no phenotype;<br>Lamin C ΔN in larval muscles:<br>• Semi-lethal or lethal depending on driver (e.g., Act5C, How24B, Mef2 = lethal; C57 = semi-lethal)<br>• Muscle nuclei: elongated, misshapen; chromatin condensation; Lamin and NPC aggregates (O-ring morphology)<br>• NE components: Otefin and Klaroid mislocalized or aggregated<br>• Cytoskeleton: absence of perinuclear microtubules, intranuclear actin rods, disrupted sarcomeric striation<br>• Adult escapers (C57 driver): twisted third legs due to impaired

			leg disc elongation<br>• Endocrine pathway: failure to induce βFtz-F1, reduced ecdysone target genes E74 and E93
[4]	GAL4-UAS mediated overexpression of wt lamin C and mutants mimicking human laminopathy Construct: pCaSpeR-hs/act (heat-shock inducible). Selected promoters: Eyeless, C57, Mef2, MHC<br><br>Mutants:<br>1. Wild-type LamC<br>2. G489V (G449V)<br>3. N496I (N456I)<br>4. V528P (L489P)<br>5. M553R (W514R)<br><br>Reference: Dialynas et al 2012	Larval bodywall muscles Larval movement<br><br>Muscle and eye disc specific	1. Normal larval movement; nuclear rim localization of lamin C and lamin Dm; normal NPC and SUN2 localization<br>2. Severe larval movement defect; extranuclear lamin C granules; cytoplasmic mislocalization of Klaroid, FG-NPCs, gp210; disrupted lamin Dm (granular pattern)<br>3. Mild larval phenotype; normal lamin C and lamin Dm localization; no cytoplasmic mislocalization of Klaroid or NPCs<br>4. Severe larval movement defect; extranuclear lamin C granules; strong cytoplasmic Klaroid and NPC mislocalization; altered LamDm pattern<br>5. Severe larval movement defect; high IF signal of lamin C; extranuclear granules; NPC and Klaroid mislocalization; altered lamin Dm signal<br><br>Viability: wt lamin C viable; 2.,3.,4.,5 were semi-lethal for C57 promoter and mostly lethal for Mef2 promoter. All were fully viable for MHC promoter
[5]	GAL4-UAS mediated over?expression of wt lamin C and mutants mimicking human laminopathy<br>Construct: pCaSpeR-hs/act (heat-shock inducible). Selected promoter: C57.<br><br>Mutants:<br>1. Wild-type LamC<br>2. G489V (G449V)<br>3. N496I (N456I)<br>4. V528P (L489P)<br>5. M553R (W514R)<br><br>Reference: Dialynas 2015	Larval bodywall muscles	1. Normal distribution of lamin C; similar nuclear strain as in mutants (except ΔN); no accumulation of redox markers; baseline expression of stress-related genes.<br>2. Lamin C distribution similar to wt; nuclear strain similar to wt; upregulation of redox stress markers CncC (NRF2 ortholog), p62/Ref(2)P, Keap1; nuclear accumulation of these proteins; activation of Nrf2/Keap1 pathway; 21 genes affected similarly as in ΔN mutant (by RNAseq).<br>3. Lamin C localization similar to wt; does not activate redox stress response; CncC and p62 not upregulated; serves as benign mutant control.<br>4. Lamin C distribution similar to wt; activation of redox stress pathway: nuclear CncC, increased p62 and Keap1 signal; moderate overlap in transcriptional profile with ΔN and G489V.<br>5. Lamin C distribution similar to wt; elevated redox stress markers (CncC, p62, Keap1); transcriptional profile overlaps partially with G489V/ΔN; nuclear strain similar to wt.<br>RNAseq analysis reported common 21 genes affected both in ΔN and G489V mutants.
[6]	GAL4-UAS mediated overexpression of wt lamin C and mutants mimicking human laminopathy Construct: pCaSpeR-hs/act (heat-shock inducible).<br><br>Mutants:<br>1. A177P,<br>2. R205W,<br>3. G489V<br>4. V528P)	Adult animals Indirect flight muscles	Fln-GAL4 driver:<br>Held-up wings in R205W, G489V, V528P<br>• Shorter sarcomeres in R205W, G489V<br>• Disrupted Z-discs and M-lines (R205W, G489V)<br>DJ694-Gal4: milder phenotypes<br>• Lamin C mutant accumulation in muscles after 3 days - higher level of lamin C mutant levels comparing to wt lamin C.<br>• Lamin C aggregates increased with age; aggregates co-localize with Ref(2)P;<br>• Cytoplasmic NPC aggregates higher after 3 weeks<br>• Nuclear blebbing, dysmorphic and misaligned nuclei.<br>• Aberrant sarcomere structure; flightless phenotype

	Selected promoters: Act88F, Fln, Flightin (during IFM sarcomere assembly); DJ694 (post-assembly) (44,45)<br><br>Reference: Chandran et al 2019		Rescue: AMPKα, dPGC-1, Thor (decreased aggregates, restored flight)<br>• No rescue: FOXO, S6K overexpression<br>• Rescue via S6K onockout: decreased aggregates, restored phenotype
[7]	GAL4-UAS mediated expression of wt lamin C and mutants mimicking human laminopathy Construct: pCaSpeR-hs/act (heat-shock inducible). Selected promoter: Hand -Cardiac specific promoter<br><br>Mutants:<br>R205W<br>G489V<br><br>Reference: Bhide et al 2018	Semi intact hearts	• Myofibrillar disorganization; enlarged and lobulated nuclei<br>• Cytoplasmic aggregates of lamin C and Otefin<br>• Elevated lamin C and Ref(2)P levels (2.5–3-fold compared to wild-type); increased number of cytoplasmic foci<br>• G489V causes milder phenotype than R205W<br>• Phenotype severity increases with age; reduced lifespan<br>• Enlarged lipid droplets in fat body; increased triglyceride levels (non-cell-autonomous effect)<br>• Nuclear accumulation of CncC (indicative of Nrf2-like redox activation)<br>• Overexpression of Atg1 reduces aggregates, restores heart structure, lowers triglyceride levels, and extends lifespan<br>• Knockdown of CncC reduces aggregates and improves heart phenotype, but does not affect fat metabolism or lifespan<br>• Best rescue achieved by combined Atg1 overexpression and CncC knockdown (restores heart, lipid profile, and lifespan)<br>• Inhibition of Atg1 worsens phenotypes; combination of Atg1 inhibition and CncC knockdown fails to rescue
[8]	GAL4-UAS-mediated expression of wt Lamin C and mutants associated with human laminopathy.<br>Construct: pUAST P-element transformation vector (Brand & Perrimon, 1993). Multiple independent lines were generated and homozygosed.<br>Selected promoter: **C57-** larval body wall muscles specific.<br><br>Mutants:<br>1. S37L,<br>2. ΔK47,<br>3. L74R,<br>4. R205W,<br>5. R237P,<br>6. G489V,<br>7. K521Q,<br>8. R564P<br>9.<br>Reference: Shaw et al., 2022	Larval body wall muscles	• Wild-type and ΔN lamin C: fully viable<br>• 1, 2, 4, 5, 7: no adult survivors<br>• 3: ~15% viability; 6 and 8: reduced adult survival<br>• wt lamin C: nuclear localization<br>• 1, 6, 7, 8: mostly cytoplasmic lamin C granules<br>• 4: lamin C blebs; NPCs nuclear, mostly in granules; chromatin and actin defects<br>• 8: granular lamin C at chromatin periphery; NPCs at nuclear rim and cytoplasmic granules<br>• NPC staining predominantly nuclear in wt (90%) and control (80%), reduced in 6 and 7 (~40%)<br>• Increased nuclear strain: 2, 3, 4<br>• Increased displacement, microtubule defects: 2, 7<br>RNAi against Koi and MSP300 did not affect nuclear strain
[9]	GAL4-UAS-mediated expression of wt lamin C and laminopathy-associated mutants.<br>Construct: lamin C cDNAs were cloned into the pUAST vector.	larval body wall muscles and adult indirect	• R264Q and R264W: sterile when expressed in larval body wall muscles<br>• R264Q and R264W: viable when expressed in indirect flight muscles (IFM); wing posture phenotype (held-out/held-up) increased with age

	Selected promoter: C57. larval body wall muscles specific.<br><br>Mutants:<br>1. R264Q,<br>2. R264W<br>3. R564P (R564P tested only in larval motility assays).<br>4.<br>Reference: Hinz et al., 2021	flight muscles (IFMs)	• R264Q and R264W: phenotype observed in ~50% of females compared to males<br>• R564P: reduced larval motility (velocity and contraction distance)<br>• wt lamin C: nuclear envelope localization with multiple granules; NPCs (mAb414) show similar pattern; lamin Dm mostly nuclear with few granules<br>• wt lamin C: microtubules appeared thickened or bead-like in regions<br>• R264Q: intranuclear ovoid lamin C structures lacking DNA; minor cytoplasmic signal near nuclei<br>• R264Q: lamin Dm clustered irregularly at NE; NPCs formed oval aggregates; microtubules disorganized<br>• R264W: elongated DAPI staining regions; fragmented lamin C at NE<br>• R264W: lamin Dm present in hemispheric NE protrusions, often granular and DNA-negative<br>• R264W: NPCs formed granules on both DNA-positive and DNA-negative regions; microtubules disrupted with linear surface deposits
[10]	GAL4-UAS-mediated expression of wt Lamin C and mutants associated with human laminopathy.<br>Construct: Lamin C transgenes were cloned into the pUAST vector containing a mini-Hsp70 promoter<br>Selected promoter:<br>C57, Hand,<br><br>Construct: P{CaSpeR-4}<br>Lsp2, r4<br>larval-fat-body-specific Gal4 drivers [P(Lsp2-Gal4.H3), stock #6357, and P[r4- Gal4}3, stock #33832, Bloomington Stock Center]<br><br>Mutants tested:<br>• K521W,<br>• R564P<br><br>Walker et al 2023	Larval body wall muscles, Fat body Cardiac muscle	• K521W and R564P: sterile<br>• R564P: wider and longer larval body wall muscles (especially segment 8); reduced larval motility<br>• R564P (cardiac muscle): adult lifespan reduced by ~50%<br><br>• wt lamin C: NE-localized, frequently granular or thickened signal with intranuclear granules<br>• wt: Otefin in intranuclear granules (no NE signal); TMEM43 at NE and as small circles<br><br>• K521W: lamin C signal concentrated in 1–2 large NE protrusions; most DAPI signal excluded from protrusion<br>• K521W: Otefin mainly at NE; TMEM43 in protrusions and partially at NE<br><br>• R564P: lamin C predominantly cytoplasmic in perinuclear aggregates<br>• R564P: Otefin at NE and cytoplasm (not overlapping lamin C); TMEM43 mostly cytoplasmic, partly at NE<br>• R564P (fat body): cytoplasmic lamin C with weak, partial NE localization

References

1. Schulze, S.R.; Curio-Penny, B.; Li, Y.; Imani, R.A.; Rydberg, L.; Geyer, P.K.; Wallrath, L.L. Molecular Genetic Analysis of the Nested Drosophila Melanogaster Lamin C Gene. *Genetics* **2005**, *171*, 185–196, doi:10.1534/genetics.105.043208.

2. Schulze, S.R.; Curio-Penny, B.; Speese, S.; Dialynas, G.; Cryderman, D.E.; McDonough, C.W.; Nalbant, D.; Petersen, M.; Budnik, V.; Geyer, P.K.; et al. A Comparative Study of Drosophila and Human A-Type Lamins. *PLoS One* **2009**, *4*, e7564, doi:10.1371/journal.pone.0007564.

3. Dialynas, G.; Speese, S.; Budnik, V.; Geyer, P.K.; Wallrath, L.L. The Role of Drosophila Lamin C in Muscle Function and Gene Expression. *Development* **2010**, *137*, 3067–3077, doi:10.1242/dev.048231.

4. Dialynas, G.; Flannery, K.M.; Zirbel, L.N.; Nagy, P.L.; Mathews, K.D.; Moore, S.A.; Wallrath, L.L. LMNA Variants Cause Cytoplasmic Distribution of Nuclear Pore Proteins in Drosophila and Human Muscle. *Hum. Mol. Genet.* **2012**, *21*, 1544–1556, doi:10.1093/hmg/ddr592.

5. Dialynas, G.; Shrestha, O.K.; Ponce, J.M.; Zwerger, M.; Thiemann, D.A.; Young, G.H.; Moore, S.A.; Yu, L.; Lammerding, J.; Wallrath, L.L. Myopathic Lamin Mutations Cause Reductive Stress and Activate the Nrf2/Keap-1 Pathway. *PLoS Genet.* **2015**, *11*, 1–21, doi:10.1371/journal.pgen.1005231.

6. Chandran, S.; Suggs, J.A.; Wang, B.J.; Han, A.; Bhide, S.; Cryderman, D.E.; Moore, S.A.; Bernstein, S.I.; Wallrath, L.L.; Melkani, G.C. Suppression of Myopathic Lamin Mutations by Muscle-Specific Activation of AMPK and Modulation of Downstream Signaling. *Hum. Mol. Genet.* **2019**, *28*, 351–371, doi:10.1093/hmg/ddy332.

7. Bhide, S.; Trujillo, A.S.; O'Connor, M.T.; Young, G.H.; Cryderman, D.E.; Chandran, S.; Nikravesh, M.; Wallrath, L.L.; Melkani, G.C. Increasing Autophagy and Blocking Nrf2 Suppress Laminopathy-Induced Age-Dependent Cardiac Dysfunction and Shortened Lifespan. *Aging Cell* **2018**, *17*, 1–14, doi:10.1111/acel.12747.

8. Shaw, N.M.; Rios-Monterrosa, J.L.; Fedorchak, G.R.; Ketterer, M.R.; Coombs, G.S.; Lammerding, J.; Wallrath, L.L. Effects of Mutant Lamins on Nucleo-Cytoskeletal Coupling in Drosophila Models of LMNA Muscular Dystrophy. *Front. Cell Dev. Biol.* **2022**, *10*, 1–18, doi:10.3389/fcell.2022.934586.

9. Hinz, B.E.; Walker, S.G.; Xiong, A.; Gogal, R.A.; Schnieders, M.J.; Wallrath, L.L. In Silico and in Vivo Analysis of Amino Acid Substitutions That Cause Laminopathies. *Int. J. Mol. Sci.* **2021**, *22*, doi:10.3390/ijms222011226.

10. Walker, S.G.; Langland, C.J.; Viles, J.; Hecker, L.A.; Wallrath, L.L. Drosophila Models Reveal Properties of Mutant Lamins That Give Rise to Distinct Diseases. *Cells* **2023**, *12*, doi:10.3390/cells12081142.
